# Hotspots sequences of *gyr*A, *gyr*B, *par*C, and *par*E genes encoded for fluoroquinolones resistance from local *Salmonella* Typhi strains in Jakarta

**DOI:** 10.1186/s12866-022-02666-z

**Published:** 2022-10-18

**Authors:** Ignes Nathania, Ita M. Nainggolan, Andi Yasmon, Angela Ch. M. Nusatia, Enty Tjoa, Wani D. Gunardi, Lucky H. Moehario

**Affiliations:** 1 Department of Microbiology, School of Medicine and Health Sciences, Atma Jaya Catholic University of Indonesia, Jalan Pluit Raya No. 2, North Jakarta, 14440 Indonesia; 2 Department of Clinical Pathology, School of Medicine and Health Sciences, Atma Jaya Catholic University of Indonesia, Jalan Pluit Raya No. 2, North Jakarta, 14440 Indonesia; 3 Eijkman Research Center for Molecular Biology, The National Research and Innovation Agency, Jalan Raya Jakarta Bogor No.46, Pakansari, Cibinong, Kabupaten Bogor, Jawa Barat 16915 Indonesia; 4 Department of Microbiology, Faculty of Medicine, Universitas Indonesia, Jalan Pegangsaan Timur 16, Jakarta, 10320 Indonesia; 5 St. Carolus Hospital, Jalan Salemba Raya No. 41, Jakarta, 10440 Indonesia; 6 Department of Microbiology, Krida Wacana Christian University, Jalan Arjuna Utara No. 6, Jakarta, 11510 Indonesia

**Keywords:** *S.* Typhi, Typhoid fever, Fluoroquinolones, Resistant, QRDR, Hotspots

## Abstract

**Background:**

Infection of *Salmonella enterica* subsp. *enterica* serovar Typhi is the primary etiology of typhoid fever globally and is common in many developing countries, especially those with dense populations and poor environmental sanitation. Antibiotic fluoroquinolones were used for the treatment in the 1980s due to the resistance to the first-line antibiotics. However, many cases of treatment failure of fluoroquinolones in typhoidal patients have been reported from numerous countries in Asia, Europe, Africa, and America. Mutations in quinolone resistance determining regions (QRDR) genes, *gyr*A, *gyr*B, *par*C, and *par*E, are found in fluoroquinolone-resistant *Salmonella* Typhi. Contrast reports came from the *S.* Typhi isolates in Indonesia, mainly Jakarta and the surroundings, obtained from patients with typhoid fever, with good sensitivity to the fluoroquinolones, i.e., nalidixic acid, ciprofloxacin, moxifloxacin, and levofloxacin. The present study, therefore, aimed to identify the hotspot sequences of *gyr*A, *gyr*B, *par*C, and *par*E genes of the local *S.* Typhi strains based on their susceptibility to fluoroquinolones from patients with typhoid fever in Jakarta and its satellite cities.

**Results:**

A total of 28 isolates were identified as *S.* Typhi. All isolates were susceptible to nalidixic acid, levofloxacin, and moxifloxacin. Twenty-seven isolates (96.4%) were susceptible to ciprofloxacin, with one isolate (3.6%) being intermediate. The hotspot sequences of *gyr*A, *gyr*B, *par*C, and *par*E genes from all isolates were identical to the fluoroquinolone-sensitive reference sequence *Salmonella enterica subsp. enterica* serovar Typhi Ty2 (NCBI GenBank AE014613.1), including the isolate with intermediate susceptibility. The mutation was not found, and amino acid deduced from all hotspots in susceptible and intermediate isolates showed no replacement in all reported codons.

**Conclusions:**

This study showed that the local *S.* Typhi strains from Jakarta and surroundings were susceptible to fluoroquinolones (nalidixic acid, ciprofloxacin, levofloxacin, and moxifloxacin), and the hotspot sequences of the *gyr*A, *gyr*B, *par*C, and *par*E genes were all identical to the reference sequence. Thus, the hotspot sequences of the *gyr*A, *gyr*B, *par*C, and *par*E genes seemingly were conserved in Jakarta’s local *S.* Typhi strains and could be considered wild type. The phenotypic susceptibility was consistent with the genotypic characteristic without non-synonymous mutations associated with drug resistance.

**Supplementary Information:**

The online version contains supplementary material available at 10.1186/s12866-022-02666-z.

## Background

Typhoid fever is a public health problem, with high morbidity and mortality rates mostly in developing countries of Africa, South Asia, and Southeast Asia [1, 2]. Infection of *Salmonella enterica* subsp. *enterica* serovar Typhi is the leading cause of the disease. People living in poverty with poor sanitation system, low water quality, and improper food handling promote higher risk of *S.* Typhi infection. It is estimated that around 13% of the world’s population lives in extreme poverty, with a poverty rate of 60% in each country [3]. Data from the Global Burden of Disease (GBD) in 2017 showed the number of *S.* Typhi infections about 10 million cases, with the number of deaths of approximately 116.800. These numbers are much higher than paratyphoid fever cases, around 3 million and 19.100 deaths [4], with the case fatality rate of typhoid fever at 10–30% without treatment and falling to 1–4% after proper treatment [2]. GBD report also revealed the Years of Life Lost (YLL) and Years Lived with Disability (YLD) of typhoid fever were around 8.332.000 and 105.500. Although the trend of typhoid fever decreased each year from 1990 to 2010, the global case still reached 13–20 million cases, with mortality number of 145.000–202.000 cases. Areas with the highest issues were South Asia, East Asia, Southeast Asia, and Sub-Saharan Africa [4].

The only way to treat typhoid fever is through antibiotic therapy. The first-line antibiotics were ampicillin, chloramphenicol, and trimethoprim-sulfamethoxazole, where chloramphenicol became the earliest antibiotic introduced in 1948 on the Malay Peninsula [5]. Ampicillin and trimethoprim-sulfamethoxazole replaced chloramphenicol therapy in the 1970s due to chloramphenicol resistance and severe side effects [5, 6]. The first report of *S.* Typhi strain resistant to chloramphenicol came from England in 1950. Later from 1962 to 1967, chloramphenicol resistance *S.* Typhi strains were also reported from India, West Africa, Greece, and Israel [7]. In 1972, MDR (*multidrug resistance*) *S.* Typhi was reported in Mexico [8]. Since the 1980s, the second-line antibiotic, fluoroquinolones, and third-generation cephalosporine have been recommended to treat MDR (*multidrug resistance*) *S.* Typhi. Alternatively, azithromycin can be applied to cure typhoid fever [9].

In recent years, multiple countries have reported the resistance and decrease-susceptibility of *S.* Typhi to fluoroquinolones. The early report came from the Middle East and Central Asia in 2014, followed by Ghana in 2016 [10, 11]. European Committee on Antimicrobial Susceptibility Testing (EUCAST) database from 2015 revealed that 6% of *Salmonella* isolates were resistant to ciprofloxacin. In 2015 as well, the U.S. National Antimicrobial Resistance Monitoring System (NARMS) announced the rising number of *Salmonella* with decreasing susceptibility to ciprofloxacin from < 0.5 to 3.5% since 1996 [9]. Hence, the antibiotic-resistant priority list published by the World Health Organization (WHO) in 2017 showed that fluoroquinolone-resistant *Salmonella* was declared in the “high” category, which meant that novel antibiotics were urgently required to be discovered as alternatives to cure typhoid patients [12]. In the meantime, a study by Moehario et al. 2019 in Indonesia showed a contrasting result that 87.5–100% of *S.* Typhi from Jakarta and Tangerang was yet sensitive to first-line antibiotics and fluoroquinolones [13]. Lugito and Cucunawangsih also revealed the similar condition in Karawaci, Tangerang, that the resistance rate of *S.* Typhi isolates to ciprofloxacin and levofloxacin was low [14]. As the capital city, Jakarta is the largest city in Indonesia and Southeast Asia, with over 10 million people inhabited. It is surrounded by satellite towns, namely Bogor, Depok, Tangerang, and Bekasi [15]. Many slums can be found around Jakarta, and these circumstances promote *S.* Typhi dissemination and infection.

Researchers have identified several point mutations in *gyr*A, *gyr*B, *par*C, and *par*E, part of the QRDR. Another mechanism that plays a role in fluoroquinolone-resistant properties is plasmid-mediated. Many plasmid-mediated quinolone resistance (PMQR) genes have been identified i.e. *qnr*A*, qnr*B*, qnr*S*, qnr*C*,* and *qnr*D [9]. A study by Koirala et al. 2012 [16] and Tack et al. 2019 [17] revealed a point mutation in *gyr*A, *gyr*B, and *par*C. According to a study in Italy by Garcia-Fernández et al. 2015, mutations were detected in *gyr*A, *gyr*B, *par*C, and *par*E [18]. Qian et al. 2020 in China also reported some mutations in all of the QRDR regions [19].

The different situation revealed in Jakarta was possibly caused by the varied length of *S.* Typhi genome. Two studies conducted by Thong et al. 1994 and 1996 have revealed different genome fragment patterns and lengths of isolates from Malaysia and Papua New Guinea using Pulsed-field Gel Electrophoresis (PFGE) and three restriction endonuclease enzymes [20]. Variation in fragment patterns and genome lengths was reported further by a study by Moehario et al. 2009 using the PFGE method. The *S.* Typhi isolates from several different areas in Indonesia appeared to have distinct genome lengths ranging from 1.495–4.561 kb and were divided into four clusters. They were from Makassar, Jakarta, Jayapura, and one cluster consisted of diverse areas [21]. On the other hand, the average genome length of > 4.300 *S.* Typhi at Typhi Pathogenwatch is 4,7 Mbp [22].

The distinct genome length, global resistance pattern, and the widespread use of antibiotics are predicted to be the main factors that induce *S.* Typhi’s resistance to antibiotics, including fluoroquinolones. The hotspot sequences of *gyr*A, *gyr*B, *par*C, and *par*E from Indonesia’s local *S.* Typhi strains have not been thoroughly studied. At the same time, most *S.* Typhi isolates in Jakarta and the neighboring towns are still susceptible to fluoroquinolones. This study aimed to examine the hotspot region of the genes encoding fluoroquinolone-resistant *gyr*A, *gyr*B, *par*C, and *par*E in the local Jakarta *S.* Typhi strains based on the fluoroquinolones susceptibility.

## Results

### Isolate identification and antibiotic susceptibility profile

A total of 28 *S.* Typhi isolates from Jakarta and its surroundings collected from 2015 to 2021 were retrieved from − 20 °C storage. These isolates originally came from patients with typhoid fever and were treated in hospitals across Jakarta and the surroundings. All of the isolates were re-identified and confirmed as *S.* Typhi. Antibiotic susceptibility from 28 isolates was determined following the guideline from CLSI 2011, CLSI 2020, and EUCAST 2022 [23–25]. All isolates (100%) were susceptible to nalidixic acid, levofloxacin, and moxifloxacin. In contrast, susceptibility to ciprofloxacin was 96.4%, and one isolate (3.6%) showed intermediate susceptibility (Table 1). None of the isolates was resistant to all of the tested fluoroquinolones.

**Table 1:** Antibiotic susceptibility profile of *S.* Typhi isolates in Jakarta

Antibiotic	Sensitive (*n* = 28)	Intermediate (*n* = 28)	Resistant (*n* = 28)
Ciprofloxacin (CLSI 2020)	27 (96.4%)	1 (3.6%)	0
Levofloxacin (EUCAST 2022)	28 (100%)	0	0
Nalidixic acid (CLSI 2011)	28 (100%)	0	0
Moxifloxacin (EUCAST 2022)	28 (100%)	0	0

### PCR amplification of *gyr*A, *gyr*B, *par*C, and *par*E

**Fig. 1:**
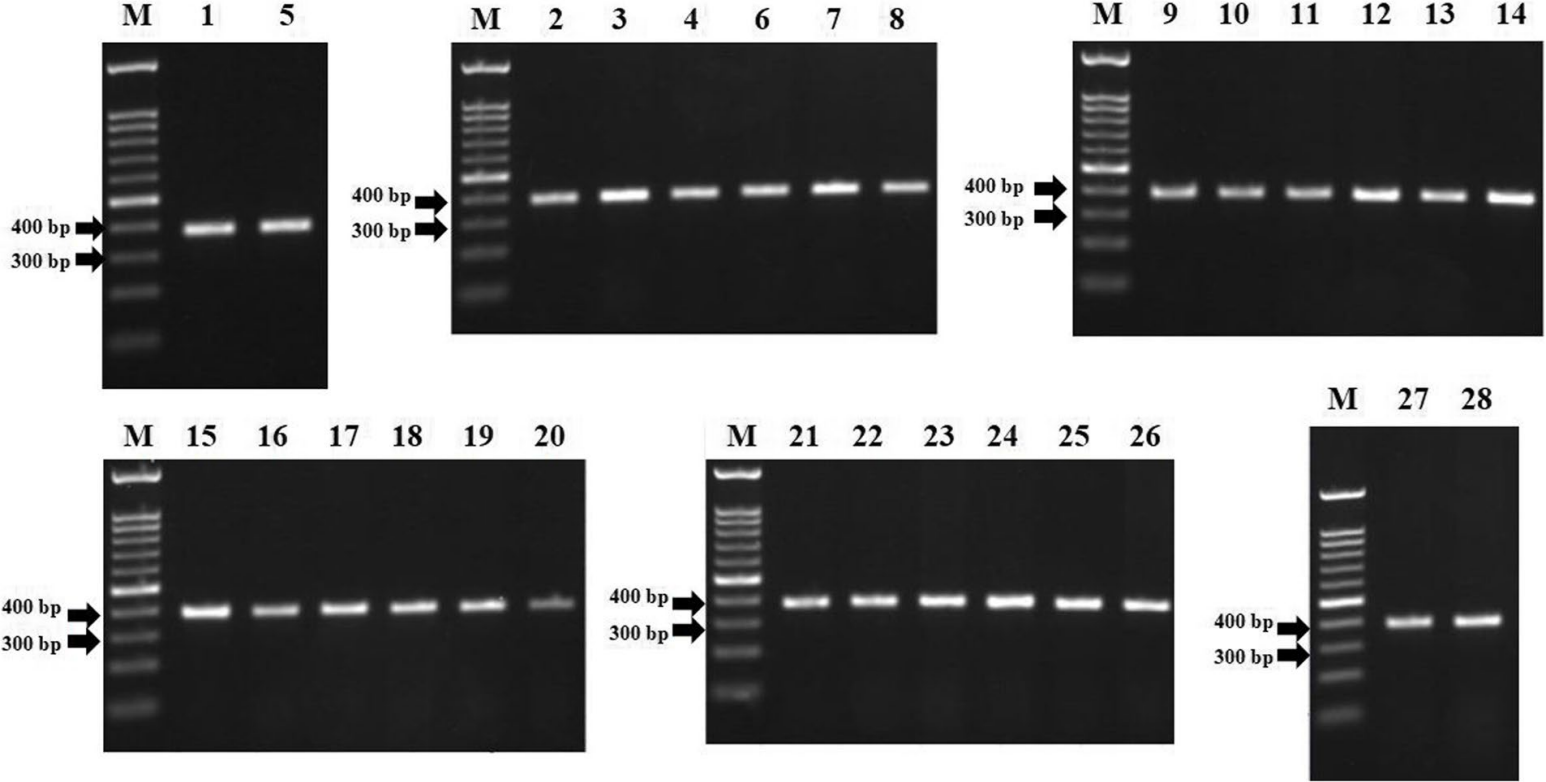
Electrophoresis of PCR product from *S.* Typhi hotspot *gyr*A. M = marker 100 bp, marker size from bottom to top (bp): 100, 200, 300, 400, 500, 600, 700, 800, 900, 1000, 1500. Numbers above the gel correspond to the isolate number in Additional file 1. All of the hotspot *gyr*A amplicons showed the expected fragments, i.e. 381 bp

**Fig. 2:**
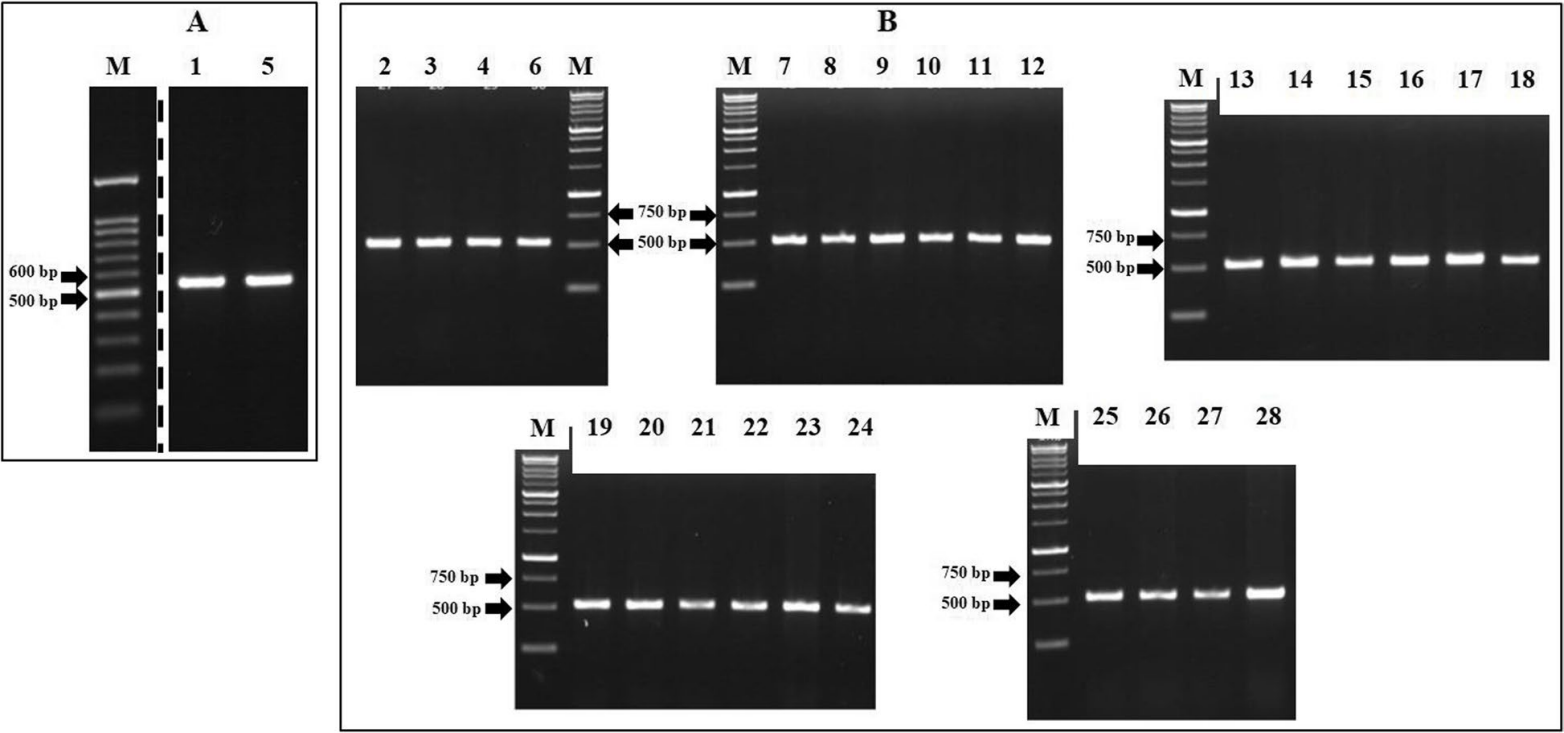
Electrophoresis of PCR product from *S.* Typhi hotspot *gyr*B. A) M = marker 100 bp, marker size from bottom to top (bp): 100, 200, 300, 400, 500, 600, 700, 800, 900, 1000, 1500. B) M = marker 1 kb, marker size from bottom to top (bp): 250, 500, 750, 1.000, 1.500, 2.000, 2.500, 3.000, 4.000, 5.000, 6.000, 8.000, 10.000. Numbers above the gel correspond to the isolate number in Additional file 1. All of the hotspot *gyr*B amplicons showed the expected fragments, i.e. 513 bp

**Fig. 3:**
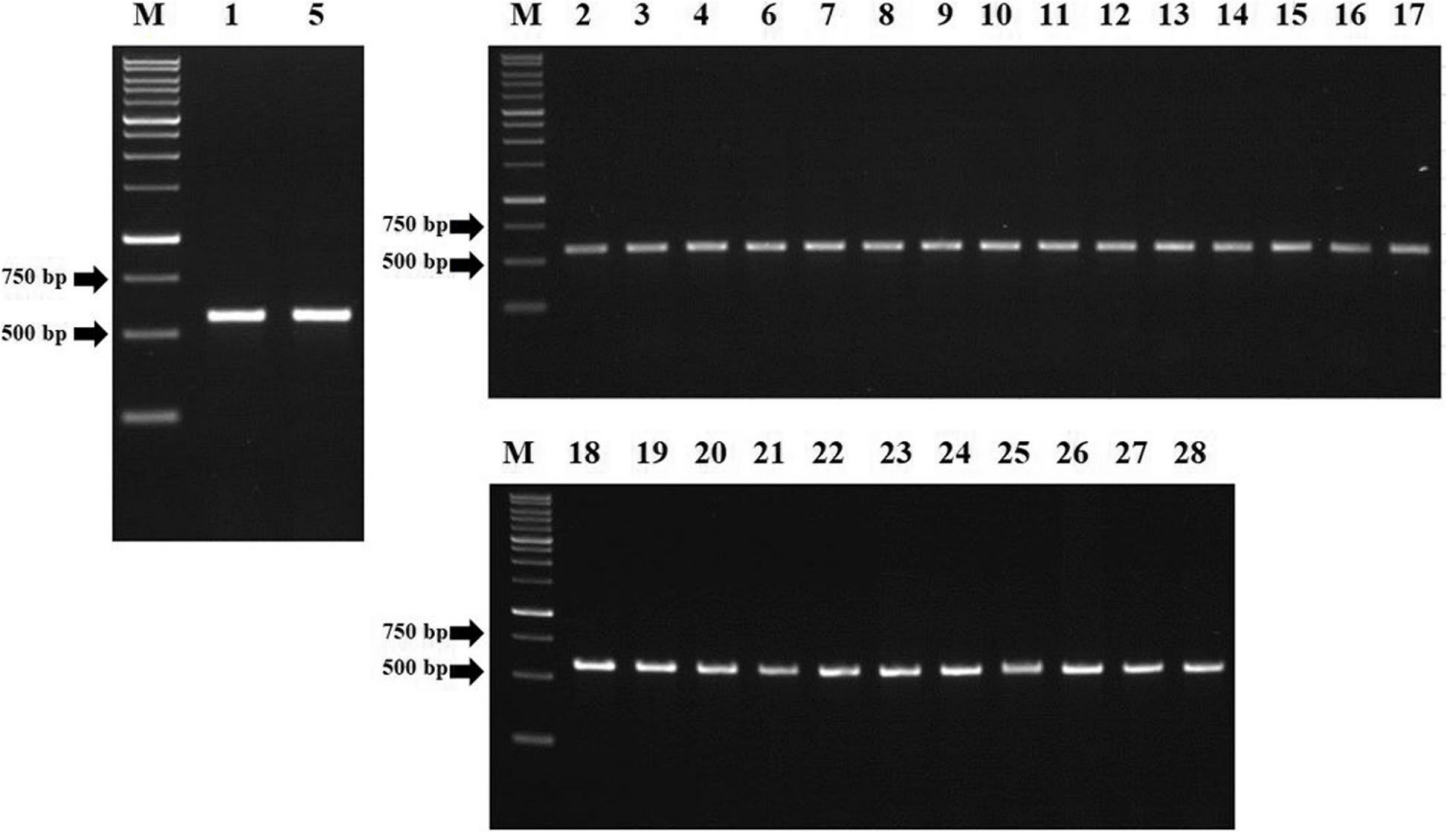
Electrophoresis of PCR product from *S.* Typhi hotspot *par*C. M = marker 1 kb, marker size from bottom to top (bp): 250, 500, 750, 1.000, 1.500, 2.000, 2.500, 3.000, 4.000, 5.000, 6.000, 8.000, 10.000. Numbers above the gel correspond to the isolate number in Additional file 1. All of the hotspot *par*C amplicons showed the expected fragments, i.e. 564 bp

**Fig. 4:**
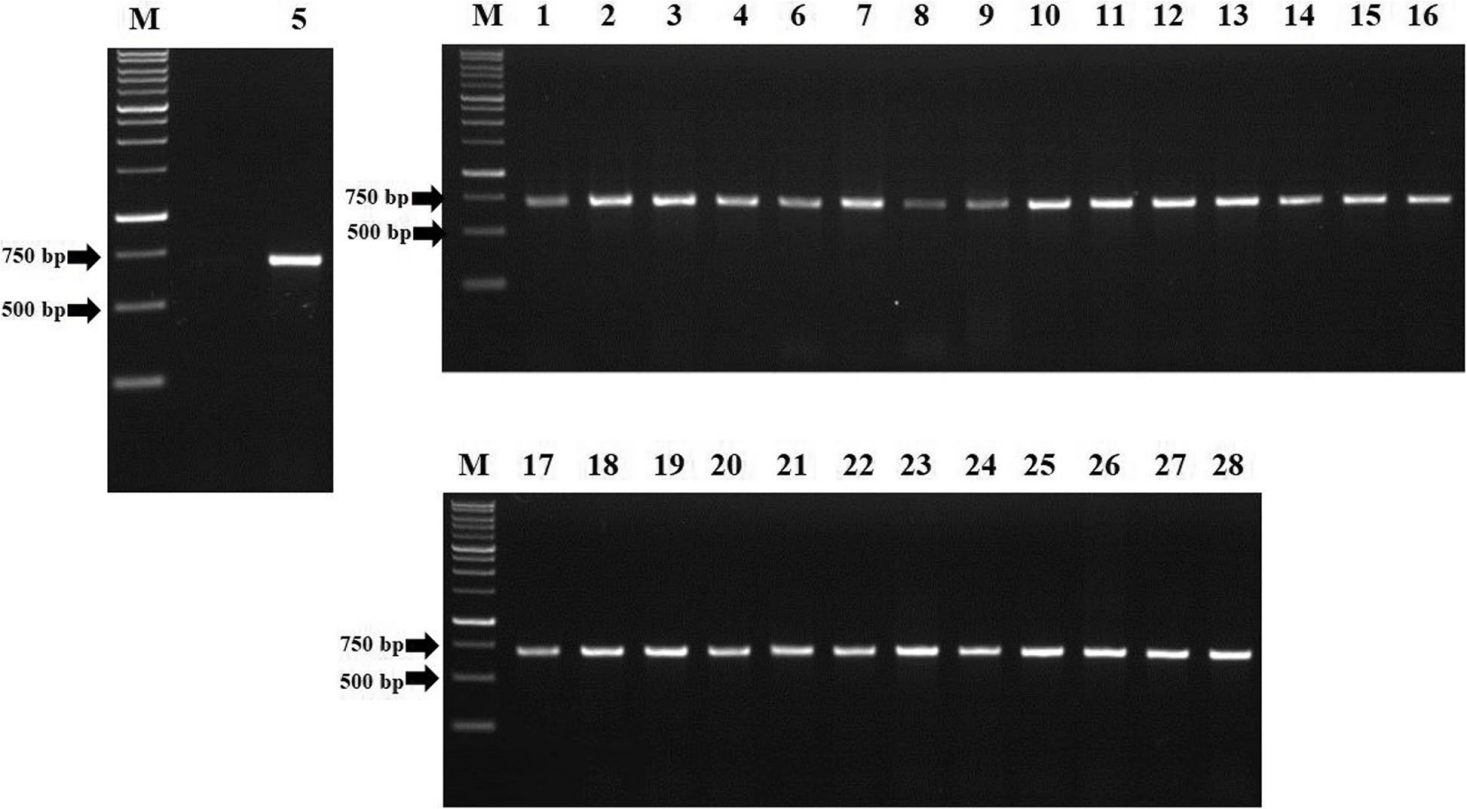
Electrophoresis of PCR product from *S.* Typhi hotspot *par*E. M = marker 1 kb, marker size from bottom to top (bp): 250, 500, 750, 1.000, 1.500, 2.000, 2.500, 3.000, 4.000, 5.000, 6.000, 8.000, 10.000. Numbers above the gel correspond to the isolate number in Additional file 1. All of the hotspot *par*E amplicons showed the expected fragments, i.e. 688 bp

The PCR amplification of the hotspot regions in *gyr*A, *gyr*B, *par*C, and *par*E as the common point mutations related to fluoroquinolones resistance showed DNA fragments that migrated at the position expected for a 381 bp, 513 bp, 564 bp, and 688 bp for each gene respectively (Fig. 1, Fig. 2, Fig. 3, Fig. 4).

### DNA sequencing and amino acid analysis

**Fig. 5:**
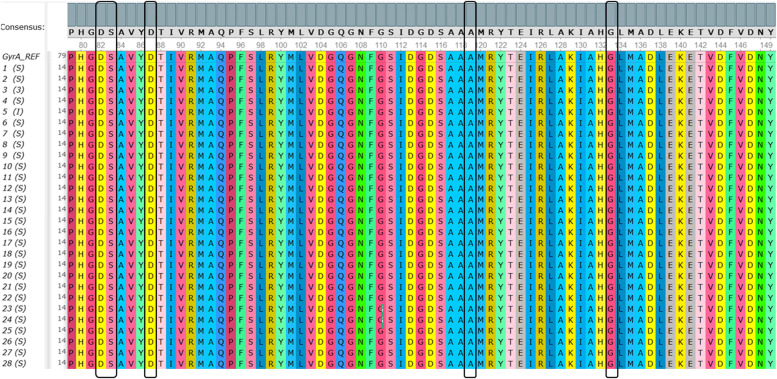
The multiple sequence alignment of hotspot *gyr*A from 28 *S.* Typhi isolates. Black boxes indicated amino acids Asp, Ser, Asp, Ala, and Gly in codons 82, 83, 87, 119, and 133, respectively. Amino acid replacements were not detected on the hotspot *gyr*A in 28 *S.* Typhi isolates. The sequence of *gyr*A can be accessed at NCBI GenBank with the accession number ON220744. Complete accession numbers are available in Additional file 1

**Fig. 6:**
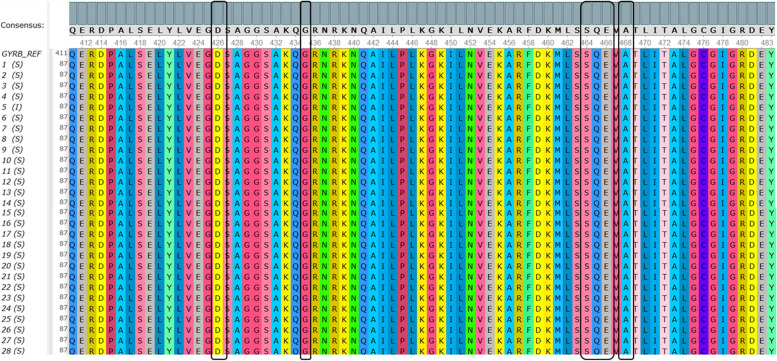
The multiple sequence alignment of hotspot *gyr*B from 28 *S.* Typhi isolates. Black boxes indicated amino acid Asp, Gly, Ser, Gln, Glu, and Ala in codons 426, 435, 464, 465, 466, and 468, respectively. Amino acid replacements were not detected on the hotspot *gyr*B in 28 *S.* Typhi isolates. The sequence of *gyr*B is available at NCBI GenBank with the accession number ON220772. Complete accession numbers are listed in Additional file 1

**Fig. 7:**
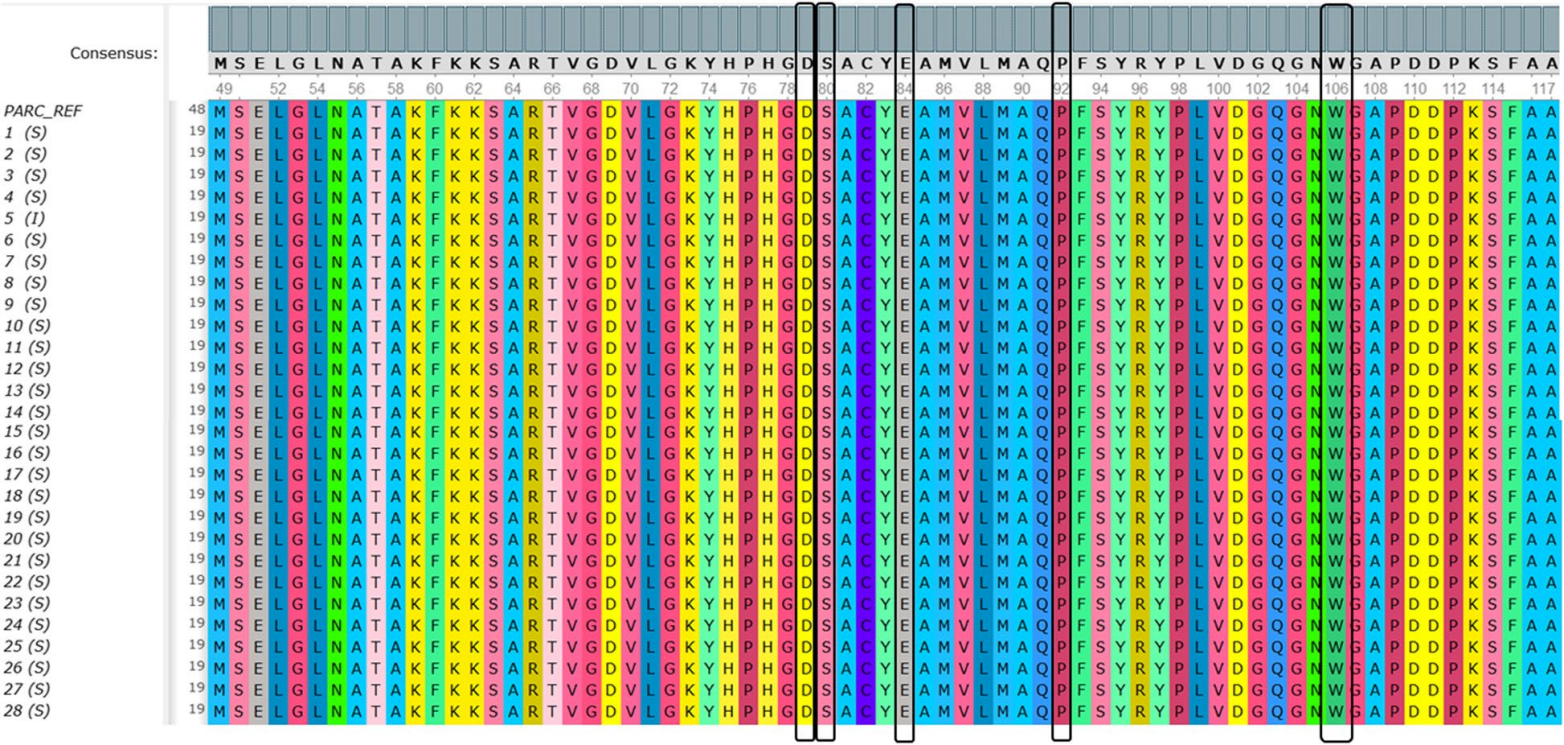
The multiple sequence alignment of hotspot *par*C from 28 *S.* Typhi isolates. Black boxes indicated amino acid Asp, Ser, Glu, Pro, and Trp in codons 79, 80, 84, 92, and 106, respectively. Amino acid replacements were not detected on the hotspot *par*C in 28 *S.* Typhi isolates. The sequence of *par*C can be accessed at NCBI GenBank with the accession number ON220800. Complete accession numbers are available in Additional file 1

**Fig. 8:**
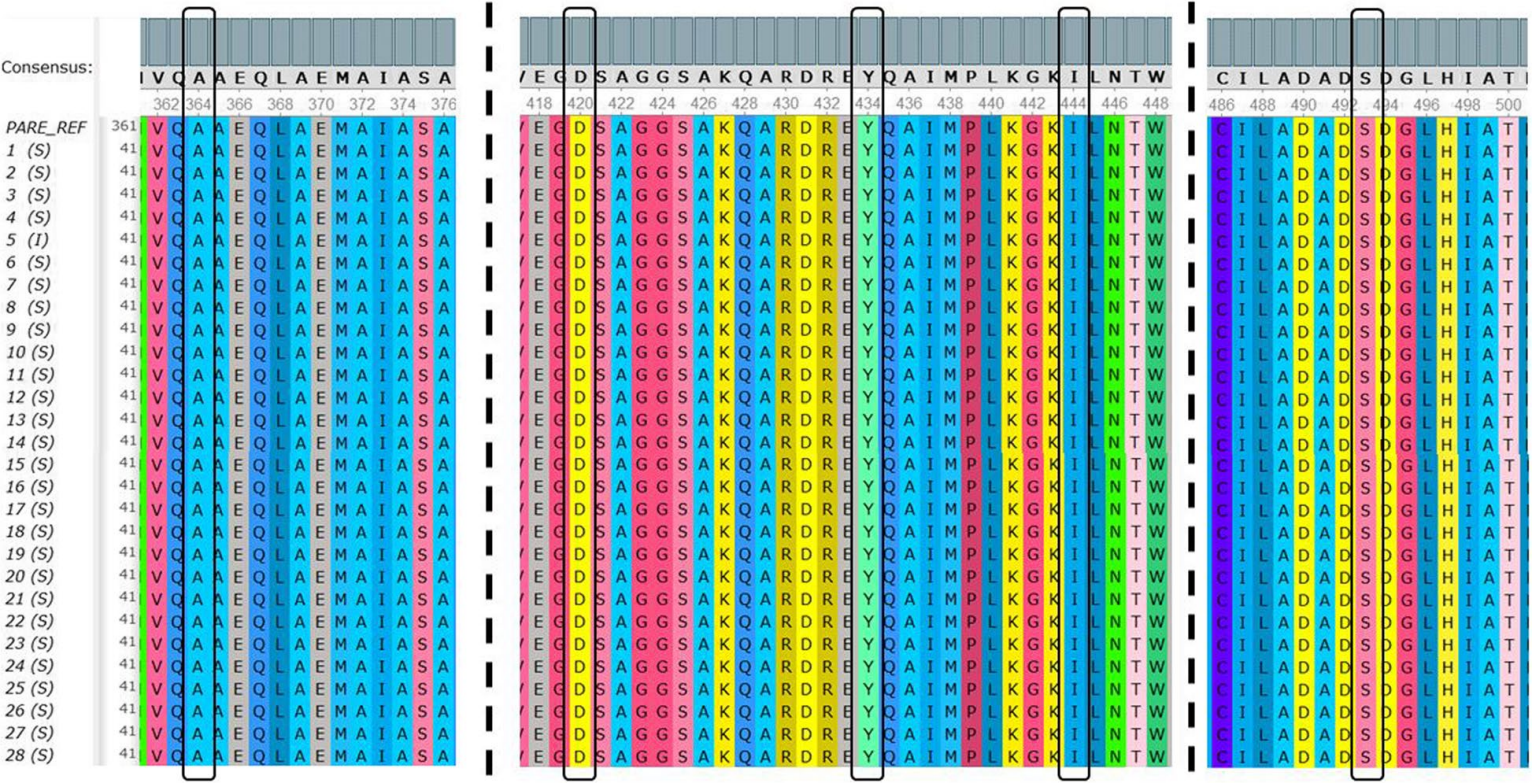
The multiple sequence alignment of hotspot *par*E from 28 *S.* Typhi isolates. Black boxes indicated amino acid Ala, Asp, Tyr, Ile, and Ser in codons 364, 420, 434, 444, and 493, respectively. Amino acid replacements were not detected on the hotspot *par*E in 28 *S.* Typhi isolates. The sequence of *par*E can be accessed at NCBI GenBank with the accession number ON220828. Complete accession numbers are available in Additional file 1. The image was cropped in order to show all of the hotspot regions in one frame

The DNA sequencing results were aligned with the reference sequence *S.* Typhi Ty2 (NCBI GenBank AE014613.1). The results showed that the hotspot *gyr*A sequences from 28 *S.* Typhi isolates were identical to the reference sequence (Fig. 5). Similar results were also found in the hotspot of *gyr*B, *par*C, and *par*E sequences (Fig. 6, Fig. 7, and Fig. 8). Point mutations in these hotspots were not detected in all genes, not even from the one isolate with intermediate ciprofloxacin susceptibility. The sequence data generated in this study have been submitted and are available at NCBI GenBank with the accession numbers listed in Additional file 1.

Codons 82, 83, 87, 119, and 133 in *gyr*A have been reported to have point mutations related to fluoroquinolones resistance. However, our results showed that the amino acid obtained from hotspot *gyr*A sequences in these codons were aspartate (Asp), serine (Ser), aspartate (Asp), alanine (Ala), glycine (Gly), respectively, in all 28 *S.* Typhi isolates, which were in accordance to the reference (Fig. 5).

In the amino acid sequences of *gyr*B, codons 426, 435, 464, 465, 466, and 468 have been reported to have point mutations related to fluoroquinolones resistance. Our study showed contrast results that the amino acid obtained from hotspot *gyr*B sequences in these codons were aspartate (Asp), glycine (Gly), serine (Ser), glutamine (Gln), glutamic acid (Glu), and alanine (Ala), respectively in all 28 *S.* Typhi isolates, which were in accordance to the reference (Fig. 6).

Similar results were also seen in *par*C. The most reported codons in *par*C with point mutations related to fluoroquinolones resistance are codon 79, 80, 84, 92, and 106. On the contrary, our results showed that the amino acid in the hotspot *par*C sequences were identical with the reference. They were aspartate (Asp), serine (Ser), glutamic acid (Glu), proline (Pro), and Tryptophan (Trp), respectively in all 28 *S.* Typhi isolates (Fig. 7).

Although mutations in *par*E related to fluoroquinolones resistance were rarely reported, some researchers have found point mutations in codons 364, 420, 434, 444, and 493. On the other hand, our results showed identical amino acids in the hotspot *par*E sequences compared to the reference, which was alanine (Ala), aspartate (Asp), tyrosine (Tyr), isoleucine (Ile), and serine (Ser), respectively, in all 28 *S.* Typhi isolates (Fig. 8).

## Discussion

Although multiple countries have announced the distribution of fluoroquinolone-resistant *S.* Typhi and the related QRDR mutations, the local *S.* Typhi strain in Indonesia, especially Jakarta, still has good susceptibility to fluoroquinolones.

Sequence mutations in the *gyr*A from fluoroquinolone-resistant *S.* Typhi have been reported from regions with and without endemic typhoid fever. Mutations frequently detected in the hotspot sequences of *gyr*A are shown in Table 2. These mutations commonly occurred and appeared to be related to the resistance to fluoroquinolones. According to the data collected from several studies, some *S.* Typhi isolates from different countries shared similar mutations in the hotspot sequences of *gyr*A. The mutation in Asp82Asn was found solely in Italy [18]. The point mutation in Ser83Phe was found in Congo [17], Italy [18], India [26–28], Nepal [16, 27], Bangladesh [29], and other South Asian countries [27]. Ser83Tyr and Asp87Asn were distributed in similar countries as Ser83Phe [17, 18, 27–29] with an additional country from China for Asp87Asn [19]. Asp87 had two other mutations, they were Asp87Val, which was found in Nepal [16], and Asp87Gly in Congo [17], China [19], and Bangladesh [29]. Mutations in Ala119Glu, Glu133Gly, and Gly133Glu were only found in Congo [17], China [19], and Iran [30], respectively. Dissimilar identification in the amino acid 133 of *gyr*A was observed in the study by Qian et al. 2020 [19] and Hamidian et al. 2011 [30]. According to the sequence data of *gyr*A *S.* Typhi from NCBI GenBank, the amino acid at position 133 is glycine instead of glutamic acid, as mentioned by Qian et al. 2020 [19]. The amino acid Glu133 was identified in *gyr*A from other *S. enterica* serotypes. Further investigation is needed to confirm the amino acid at position 133. In contrast to the stated reports above, our study showed that all hotspot *gyr*A regions had identical sequences with the reference NCBI GenBank (AE014613.1) at codon Asp82, Ser83, Asp 87, Ala119, and Gly133. Thus, amino acid replacements were not detected in all susceptible and ciprofloxacin intermediate *S.* Typhi isolates.

**Table 2:** Mutations found in the hotspot *gyr*A of *S.* Typhi

Mutation	Susceptibility Profile	Study
**Asp82Asn**	NAL (R)	[18]
**Ser83Phe**	OFX (R), CIP (R), NAL (R)	[16] ^f^
	CIP (DS)	[17]
	CIP (R), NAL (R)	[18]
	NAL (I) CIP (S)	[18]
	CIP (R), NAL (R)	[18] ^a,e^
	NAL (I) CIP (S)	[18] ^b^
	NAL (R), CIP (S)	[19]^n^
	NAL (R), CIP (I)	[19]^n,o,p^
	CIP (R), NAL (R)	[26]
	OFX (R), CIP (R), NAL (R)	[27] ^g^
	CIP (DS), NAL (R)	[28]
	CIP (R), NAL (R)	[28] ^j^
	CIP (DS), NAL (R)	[29] ^k^
	CIP (R), NAL (R)	[29]^m^
**Ser83Tyr**	CIP (DS)	[17]
	NAL (I), CIP (S)	[18] ^c^
	NAL (R), CIP (I)	[19]
	NAL (R), CIP (S)	[19]
	CIP (R), NAL (R)	[19]^q^
	OFX (R), CIP (R), NAL (R)	[27] ^h^
	CIP (DS), NAL (R)	[28]
	CIP (DS), NAL (R)	[29] ^k^
	CIP (R), NAL (R)	[29] ^l^
**Asp87Asn**	CIP (R), NAL (R)	[19]
	NAL (R), CIP (I)	[19] ^r^
	NAL (R), CIP (S)	[19] ^r,s^
	CIP (R), NAL (R)	[19] ^s^
	CIP (R), NAL (R)	[18] ^d,e^
	NAL (I) CIP (S)	[18] ^b^
	CIP (R), NAL (R)	[26]
	OFX (R), CIP (R), NAL (R)	[27] ^i^
	CIP (R), NAL (R)	[28] ^j^
	CIP (DS), NAL (R)	[29] ^k^
**Asp87Val**	OFX (R), CIP (R), NAL (R)	[16] ^f^
**Asp87Gly**	CIP (DS)	[17]
	NAL (R), CIP (I)	[19] ^t^
	CIP (R), NAL (R)	[19] ^t^
	CIP (R), NAL (R)	[29] ^m^
**Ala119Glu**	CIP (DS)	[17]
**Glu133Gly**	NAL (R), CIP (S)	[19] ^n,r,u^
	NAL (R), CIP (I)	[19]^n,o,p,r,t^
	CIP (R), NAL (R)	[19] ^q,t^
**Gly133Glu**	NAL (DS)	[30]^v^

^a^Double mutations: *gyr*A Ser83Phe and *gyr*B Gly435Ala; *gyr*A Ser83Phe and *gyr*B Gly435Glu; *gyr*A Ser83Phe and *gyr*B Gly435Val

^b^Four mutations in one isolate: *gyr*A Ser83Phe, *gyr*A Asp87Asn, *gyr*B Gly435Glu, and *par*C Ser80Ile

^c^Double mutations in one isolate: *gyr*A Ser83Tyr and *par*E Ser493Phe

^d^Double mutations in one isolate: *gyr*A Asp87Asn and *gyr*B Gly435Glu

^e^Three mutations in one isolate: *gyr*A Ser83Phe, *gyr*A Asp87Asn, and *par*C Ser80Ile

^f^Three mutations: *gyr*A Ser83Phe, *gyr*A Asp87Val, and *par*C Ser80Ile

^g^Three mutations: *gyr*A Ser83Phe, *gyr*A Asp87Val, and *par*C Ser80Ile; Double mutations: *gyr*A Ser83Phe and *par*C Glu84Gly; *gyr*A Ser83Phe and *par*E Ala364Val, in some isolates

^h^Double mutations: *gyr*A Ser83Tyr and *par*E Ala364Val

^i^Double mutations: *gyr*A Asp87Asn and *par*E Ala364Val

^j^Double mutations: *gyr*A Ser83Phe and *gyr*A Asp87Asn

^k^Single mutation with increased efflux pump activity for nalidixic acid

^l^Single mutation with the presence of *qnr*S and increased efflux pump activity for both nalidixic acid and ciprofloxacin

^m^Three mutations: *gyr*A Ser83Phe, *gyr*A Asp87Gly, *par*C Glu92Lys, and increased efflux pump activity for nalidixic acid

^n^Double mutations: *gyr*A Ser83Phe and *gyr*A Glu133Gly

^o^Three mutations: *gyr*A Ser83Phe, *gyr*A Glu133Gly, and *par*E Ile444Ser

^p^Four mutations: *gyr*A Ser83Phe, *gyr*A Glu133Gly, *par*E Ile444Ser, and *par*E Tyr434Ser

^q^Four mutations: *gyr*A Ser83Tyr, *gyr*A Asp87Asn, *gyr*A Glu133Gly, and *par*C Glu84Lys

^r^Double mutations: *gyr*A Asp87Asn and *gyr*A Glu133Gly

^s^Single mutations *gyr*A Asp87Asn and the presence of *aac(6′)-ib-cr4*

^t^Double mutations: *gyr*A Asp87Gly and *gyr*A Glu133Gly

^u^Double mutations: *gyr*A Glu133Gly and *gyr*B Ser426Gly

^v^Double mutations: *gyr*A Ser83Phe and *gyr*A Gly133Glu

A similar situation also occurred in *gyr*B, another QRDR gene. Gly435 had three mutations, Gly435Ala, Gly435Glu, and Gly435Val, that were found in Italy. These isolates were from patients with travel histories to Bangladesh and India. Other isolates were isolated from Italian residents that came from Italian, India, and Bangladesh [18]. In Congo and France, the researcher observed mutation in Ser464Tyr [17, 31]. In the Indian subcontinent, Ser464Phe was reported by Britto et al. 2020 [27]. Despite mutations in amino acids Gln 465Leu, Glu466Asp, and Ala 468Glu, some *S.* Typhi isolates in France showed an interesting profile that was pansusceptible, including fluoroquinolones [31]. Meanwhile, in Congo, Glu466Asp was identified in isolates with decreased susceptibility to ciprofloxacin [17]. Ser426Gly was detected in *S.* Typhi isolates from China only [19]. However, according to the current survey, the amino acid at position 426 was found to be an aspartic acid. Further investigation is needed to confirm the serine amino acid at position 426 of *gyr*B. Although many reports on mutations have been reported earlier, we detected the contrary results from the current study with no point mutations at the hotspot *gyr*B regions, which were Asp426, Gly435, Ser464, Gln465, Glu466, and Ala468. All of them were consistent with the reference sequences. The mutations identified in the hotspot sequences of *gyr*B earlier are summarized in Table 3.

**Table 3:** Mutations found in the hotspot *gyr*B of *S.* Typhi

Mutation	Susceptibility Profile	Study
**Ser426Gly**	CIP (S), NAL (R)	[19] ^1^
**Gly435Ala**	NAL (R), CIP (R)	[18] ^a^
**Gly435Glu**	NAL (R) CIP (R), NAL (I) CIP (S)	[18] ^b^
**Gly435Val**	NAL (R), CIP (R)	[18] ^c^
**Ser464Tyr**	CIP (DS)	[17]
	NAL (S), CIP (DS)	[31]
**Ser464Phe**	OFX (R), CIP (R), NAL (R)	[27]
**Gln465Leu**	Pansusceptible	[31]
**Glu466Asp**	CIP (DS)	[17]
	Pansusceptible	[31]
**Ala468Glu**	Pansusceptible	[31]

^1^Double mutations: *gyr*A Glu133Gly and *gyr*B Ser426Gly

^a^Double mutations: *gyr*A Ser83Phe and *gyr*B Gly435Ala

^b^Double mutations: *gyr*A Asp87Asn and *gyr*B Gly435Glu; *gyr*A Ser83Phe and *gyr*B Gly435Glu; Four mutations in one isolate: *gyr*A Ser83Phe, *gyr*A Asp87Asn, *gyr*B Gly435Glu, and *par*C Ser80Ile

^c^Double mutations: *gyr*A Ser83Phe and *gyr*B Gly435Val

Sequence mutations related to fluoroquinolone-resistant *S.* Typhi in *par*C gene have also been reported, although they were less common than *gyr*A and *gyr*B. Table 4 showed the mutations detected in the hotspot sequences of *par*C.

**Table 4:** Mutations found in the hotspot *par*C of *S.* Typhi

Mutation	Susceptibility Profile	Study
**Asp79Gly**	NAL (R) CIP (S)	[19]
**Ser80Ile**	OFX (R), CIP (R), NAL (R)	[16]^1^
**Glu84Lys**	CIP (R), NAL (R)	[19]^a^
**Glu84Gly**	OFX (R), CIP (R), NAL (R)	[27]^b^
**Glu92Lys**	NAL (R) CIP (R), NAL (I) CIP (S)	[29] ^c^
**Trp106Gly**	NAL (R), CIP (R)	[26]

^1^Three mutations: *gyr*A Ser83Phe, *gyr*A Asp87Val, and *par*C Ser80Ile

^a^Four mutations: *gyr*A Ser83Tyr, *gyr*A Asp87Asn, *gyr*A Glu133Gly, and *par*C Glu84Lys

^b^Double mutations: *gyr*A Ser83Phe and *par*C Glu84Gly

^c^Three mutations: *gyr*A Ser83Phe, *gyr*A Asp87Gly, *par*C Glu92Lys, and increased efflux pump activity for nalidixic acid

Only five mutations have been identified in *par*C. A point mutation in the amino acid Asp79Gly was found in China from isolates resistant to nalidixic acid but susceptible to ciprofloxacin, as reported by Qian et al. 2020. They also detected mutation in Glu84Lys from isolates resistant to ciprofloxacin and nalidixic acid [19]. In Nepal, Koirala et al. 2012 identified a mutation in Ser80Ile from isolate resistant to ofloxacin, ciprofloxacin, and nalidixic acid [16]. Chiou et al. 2014 found a mutation in Glu92Lys from an isolate originating from Bangladesh [29]. The glutamic acid at position 92 should be examined since the amino acid proline was considered at this codon. Gopal et al. 2016 showed a mutation in Trp106Gly of *par*C in *S.* Typhi isolate from India resistant to ciprofloxacin and nalidixic acid [26]. The hotspot *par*C regions of all *S.* Typhi isolates from the current study were in line with the reference, in which the codons 79, 80, 84, 92, and 106 were aspartate, serine, glutamic acid, proline, and tryptophan, respectively.

The mutations in *gyr*A, *gyr*B, and *par*C have been mainly reported as the reason for quinolone resistance in *S. *Typhi. On the other hand, only a few reports mentioned the mutations in *par*E regions. The list of reported mutations in hotspot *par*E is shown in Table 5. Accou-Demartin et al. 2011 in France observed mutation at Asp420Asn in *par*E from isolates resistant to nalidixic acid but susceptible to ciprofloxacin [31]. Garcia-Fernández et al. 2015 in Italy reported a mutation in amino acid Ser493Phe in *S.* Typhi isolated from a patient that traveled to India with resistance pattern intermediate susceptibility to nalidixic acid but susceptible to ciprofloxacin [18]. Two mutations in Ile444Ser, and Tyr434Ser of *par*E was found in China by Qian et al. 2020 from *S.* Typhi isolates resistant to nalidixic acid and intermediate susceptibility to ciprofloxacin [19]. In India, Britto et al. 2020 identified one mutation in *par*E Ala364Val from isolates resistant to ciprofloxacin, ofloxacin, and nalidixic acid [27]. Contrastingly, the present study showed amino acids in hotspots *par*E were Ala64, Asp420, Tyr434, Ile444, and Ser493, which were identical to the reference NCBI GenBank (AE014613.1), both in susceptible and intermediate susceptibility isolates to ciprofloxacin.

**Table 5:** Mutations found in the hotspot *par*E of *S.* Typhi

Mutation	Susceptibility Profile	Study
**Ala364Val**	OFX (R), CIP (R), NAL (R)	[27]^a^
**Asp420Asn**	CIP (S), NAL (R)	[31]^b^
**Tyr434Ser**	CIP (I), NAL (R)	[19] ^c^
**Ile444Ser**	CIP (I), NAL (R)	[19] ^c,d^
**Ser493Phe**	NAL (I), CIP (S)	[18]^e^

^a^Double mutations: *gyr*A Ser83Phe and *par*E Ala364Val; *gyr*A Ser83Tyr and *par*E Ala364Val; *gyr*A Asp87Asn and *par*E Ala364Val

^b^Double mutations: *gyr*A Ser83Phe and *par*E Asp420Asn

^c^Four mutations: *gyr*A Ser83Phe, *gyr*A Glu133Gly, *par*E Ile444Ser, and *par*E Tyr434Ser

^d^Three mutations: *gyr*A Ser83Phe, *gyr*A Glu133Gly, and *par*E Ile444Ser

^e^Double mutations in one isolate: *gyr*A Ser83Tyr and *par*E Ser493Phe

Multiple research across Indonesia supported our results on the susceptibility profile of the local *S.* Typhi strains in Jakarta. *S.* Typhi isolated from 2002 to 2008 in Jakarta and its surroundings showed good susceptibility to ciprofloxacin that was about two decades ago [32] and interestingly a similar profile of susceptibility was found in *S.* Typhi isolated in the late 2010s to 2020s [13, 14]. However, *S.* Typhi isolates collected in Sulawesi, an island located in the East of Indonesia, showed an increasing number of *S.* Typhi resistant to ciprofloxacin [33], and ofloxacin [34].

The distinct strain of *S.* Typhi could be another possible reason for the difference in the susceptibility profile of *S.* Typhi isolates from Jakarta. The study carried out by Chiou et al. 2014 about the genetic relationship of *S.* Typhi from Taiwan, Indonesia, Vietnam, and Bangladesh showed that antibiotic susceptibility is related to clonal variation. Isolates from Bangladesh and Vietnam were closely related with high resistance to nalidixic acid and ciprofloxacin. Isolates from Indonesia were closely related to those from Taiwan and were distant from isolates from Bangladesh and Vietnam. Almost all isolates from Indonesia and Taiwan were sensitive to fluoroquinolones, with a low resistance to nalidixic acid [29]. In 2019, Wang et al. also reported that isolates from Indonesia shared the same genotype as the indigenous strain in Taiwan, which was in agreement with the study by Chiou et al. 2014. All isolates in the clade have good susceptibility to ciprofloxacin [35]. Further, the study by Ingle et al. 2019 found three clonalities of *S.* Typhi isolates from Indonesia. One clonality shared the same genotype with isolates from the Philippines, France, and the USA, while the other was also detected in Peru and Bangladesh. All of the *S.* Typhi isolates in these two clonalities were sensitive to fluoroquinolones and had no QRDR mutations, except the isolates from Bangladesh and Peru had a mutation in *gyr*A [36]. Baker et al. 2008 also reported that no DNA gyrase mutations were detected in *S.* Typhi isolates from Indonesia, although fluoroquinolone-resistant H58 strains have been introduced from neighboring countries [37].

Fluoroquinolones target and convert topoisomerase IV and DNA gyrase. The binding of the antibiotics to both DNA-enzyme complexes thus inhibits the enzyme activity [38]. Mutation in QRDR results in disturbance in replication and separation of DNA, which ultimately will cause cell lysis and cell death [39]. Other mechanisms of fluoroquinolones resistance in bacteria are mediated by plasmids, the so-called PMQR, and chromosome-mediated efflux pump resistance. The expression of PMQR genes has been reported by Qian et al. 2020 through the existence of *aac(6*′*)-ib-cr-4*, *qnrS1* and *qnrB4* in some *S.* Typhi isolates that were resistant to nalidixic acid and ciprofloxacin [18]. According to Ingle et al. 2019, there were no PMQR genes reported in *S.* Typhi isolates from Indonesia [36]. The existence of PMQR genes in Indonesia has not been reported yet and there are limited reports about the role of efflux pump in fluoroquinolone-resistant *S.* Typhi as well.

## Conclusion

Despite surging cases and mutations in QRDR genes of fluoroquinolone-resistant *S.* Typhi reported worldwide, the overall local strain *S.* Typhi in Jakarta and surroundings showed good susceptibility to fluoroquinolones. Point mutations were not detected in the hotspot *gyr*A, *gyr*B, *par*C, and *par*E areas of all isolates, and no amino acid replacements in all reported codons. All DNA sequences from this study were identical to the reference sequence *S.* Typhi Ty2 (NCBI GenBank AE014613.1). The absence of point mutations in all hotspots indicated that the phenotypic profile of *S.* Typhi local strains in their susceptibility to ciprofloxacin was consistent with the genotypic characteristic. More research on PMQR genes and efflux pump might be worth to find out the alternative resistance mechanism on intermediate resistance isolate from this research. Further study on resistant local strains *S.* Typhi, if any, would shed more information about mechanisms of resistance to fluoroquinolones.

## Methods

This research was conducted in the Laboratory of Microbiology and Biomolecular, School of Medicine and Health Sciences, Atma Jaya Catholic University of Indonesia. This study was approved by Ethical Committee of School of Medicine and Health Science, Atma Jaya Catholic University of Indonesia (Ethical approval number: 08/03/KEP-FKIKUAJ/2021 and 9/08/KEP-FKIKUAJ/2021).

### Specimen collection and bacterial identification

In this study, 28 *S.* Typhi isolates from 2015 to 2021 were used. Most of them were recovered from the storage of the Laboratory of Microbiology, School of Medicine and Health Sciences, Atma Jaya Catholic University of Indonesia. Two isolates were received from hospitals in Jakarta and surroundings. Identification of *S.* Typhi was performed previously using Microbact 12A (Oxoid®) and standard microbiological procedures. Isolates were inoculated in Brain Heart Infusion Broth (BHIB) and Blood Agar (BA). Biochemical test was performed with Triple sugar iron agar (TSIA) and Xylose-lysine-deoxycholate agars (XLD). Positive *S.* Typhi isolate was characterized by the presence of black precipitate and black-centered colony, respectively. Isolates were further identified using Gram staining and serotyping by slide agglutination test with commercial antiserum O9 and antiserum O12 (Difco®). According to the White-Kauffmann Le-Minor scheme classification, *S.* Typhi was identified with positive agglutination. Isolates were then preserved on Nutrient Agar (NA) slant at room temperature. All inoculated media were incubated for 24 h at 35 °C.

### Antibiotic susceptibility test

Antibiotic susceptibility test for ciprofloxacin 5 μg (CIP), nalidixic acid 30 μg (NAL), levofloxacin 5 μg (LVX), and moxifloxacin 5 μg (MXF) was performed with Kirby Bauer or disk diffusion method on Mueller-Hinton agar according to the guideline from Clinical and Laboratory Standards Institute CLSI M100 [23]. The breakpoints of nalidixic acid and ciprofloxacin adhered to the standard from CLSI 2011 and CLSI 2020, respectively [23, 24]. Meanwhile, the breakpoints of levofloxacin and moxifloxacin complied with the data from EUCAST 2022 [25]. *Escherichia coli* ATCC 25922 was used as the reference for the antibiotic susceptibility test.

### DNA extraction

The genomic DNA from all isolates was extracted using QIAmp DNA Mini Kit (Qiagen®) from overnight culture on NA slant according to the instruction from the kit. The extracted DNA was stored in the elution buffer provided by the kit and placed at − 20 °C. The presence of the genomic DNA was confirmed by electrophoresis in 0.5% (w/v) agarose gel at 90 V for 60–90 minutes.

### Amplification of *gyr*A, *gyr*B, *par*C, and *par*E

The hotspot regions (*gyr*A, *gyr*B, *par*C, and *par*E) listed in Table 2 – Table 5 were amplified by polymerase chain reaction (PCR) using the primers shown in Table 6. These primers were specifically designed for this research according to the reference sequences from NCBI GenBank *S.* Typhi Ty2 (AE014613.1). Hotspot regions were chosen based on the frequent mutations related to fluoroquinolones resistance. PCR was performed using *Taq* PCR Master Mix Kit (Qiagen^®^) as specified by the manufacturer’s manual with a final reaction volume of 50 μl containing 8 μl (≤1 μg/reaction) of DNA template. The annealing temperature was 60 °C followed by 35x cycles and had been optimized prior to the PCR amplification.

**Table 6:** Primer sequences of *gyr*A, *gyr*B, *par*C, and *par*E

Genes		Size (bp)	Sequences
*gyrA*	F	381	5′- AAAAATCTGCCCGTGTCGTTG-3′
	R		5′- TCACTTCCGTCAGGTTGTGC-3′
*gyrB*	F	513	5′- AGGTCTGATTGCGGTGGTTT −3′
	R		5′- AGCTTGTCCGGGTTGTACTC −3′
*parC*	F	564	5′-GATCATGGATCGTGCGTTGC-3′
	R		5′-GGCCCCTGAACGATATCCAG-3′
*parE*	F	688	5′- CGCTTATGTGCTCTCCGTG −3′
	R		5′- CGCCTTCTCTTCTTCCGTCA −3′

The presence of the targeted fragments was confirmed by electrophoresis, with 4 μl of the PCR product and 1 μl of loading dye loaded in 1.5% (w/v) agarose gel submerged in 1x Tris Boric acid EDTA (TBE). The gel was run at 90 V for 60–90 minutes and stained with Florosafe (1st Base®). The DNA Ladder (Geneaid^®^) was used to determine the PCR product’s molecular size. The DNA fragment was visualized by gel documentation system (Biorad^®^). Confirmation of gel electrophoresis results was done by 1st Base^®^.

### DNA sequencing

All PCR products were sequenced by the 1st Base^®^, Malaysia, using the Sanger sequencing method. SeqTrace and Unipro UGENE were used to analyze the sequences, and the results were compared to the reference sequence (NCBI GenBank AE014613.1).

## Supplementary Information

**Additional file 1: Table S1.** NCBI Genbank accession numbers and other information related to all hotspot sequences from *S.* Typhi isolates in Jakarta. **Table S2.** Electrophoresis of PCR product from *S.* Typhi hotspot *gyr*A and *gyr*B with the expected fragments of 381 bp and 513 bp, respectively. **Figure S1.** Original electrophoresis image of PCR product from *S.* Typhi hotspot *gyr*A and *gyr*B with the expected fragments of 381 bp and 513 bp, respectively. **Table S3.** Electrophoresis of PCR product from *S.* Typhi hotspot *gyr*A and *gyr*B with the expected fragments of 381 bp and 513 bp, respectively. **Figure S2.** Original electrophoresis image of PCR product from *S.* Typhi hotspot *gyr*A and *gyr*B with the expected fragments of 381 bp and 513 bp, respectively. **Table S4.** Electrophoresis of PCR product from *S.* Typhi hotspot *par*C with the expected fragments of 564 bp. **Figure S3.** Original electrophoresis image of PCR product from *S.* Typhi hotspot *par*C with the expected fragments of 564 bp. **Table S5.** Electrophoresis of PCR product from *S.* Typhi hotspot *par*C with the expected fragments of 564 bp. **Figure S4.** Original electrophoresis image of PCR product from *S.* Typhi hotspot *par*C with the expected fragments of 564 bp. **Table S6.** Electrophoresis of PCR product from *S.* Typhi hotspot *par*E with the expected fragments of 688 bp. **Figure S5.** Original electrophoresis image of PCR product from *S.* Typhi hotspot *par*E with the expected fragments of 688 bp. **Table S7.** Electrophoresis of PCR product from *S.* Typhi hotspot *par*E with the expected fragments of 688 bp. **Figure S6.** Original electrophoresis image of PCR product from *S.* Typhi hotspot *par*E with the expected fragments of 688 bp.

## Data Availability

All data generated during this study are included in this published article and available in NCBI Genbank with the links provided in the supplementary information files.

## Abbreviations

S: Sensitive
I: Intermediate
R: Resistant
DC: Decreased susceptibility
