# Supplementary material for: Hotspots sequences of gyrA, gyrB, parC, and parE genes encoded for fluoroquinolones resistance from local Salmonella Typhi strains in Jakarta
Source: BMC Microbiol. 2022 Oct 18;22:250. doi: 10.1186/s12866-022-02666-z (PMC9578181; doi:10.1186/s12866-022-02666-z)
Supplement: Supplementary file 1 — Additional file 1: Table S1. NCBI Genbank accession numbers and other information related to all hotspot sequences from S. Typhi isolates in Jakarta. Table S2. Electrophoresis of PCR product from S. Typhi hotspot gyrA and gyrB with the expected fragments of 381 bp and 513 bp, respectively. Figure S1. Original electrophoresis image of PCR product from S. Typhi hotspot gyrA and gyrB with the expected fragments of 381 bp and 513 bp, respectively. Table S3. Electrophoresis of PCR product from S. Typhi hotspot gyrA and gyrB with the expected fragments of 381 bp and 513 bp, respectively. Figure S2. Original electrophoresis image of PCR product from S. Typhi hotspot gyrA and gyrB with the expected fragments of 381 bp and 513 bp, respectively. Table S4. Electrophoresis of PCR product from S. Typhi hotspot parC with the expected fragments of 564 bp. Figure S3. Original electrophoresis image of PCR product from S. Typhi hotspot parC with the expected fragments of 564 bp. Table S5. Electrophoresis of PCR product from S. Typhi hotspot parC with the expected fragments of 564 bp. Figure S4. Original electrophoresis image of PCR product from S. Typhi hotspot parC with the expected fragments of 564 bp. Table S6. Electrophoresis of PCR product from S. Typhi hotspot parE with the expected fragments of 688 bp. Figure S5. Original electrophoresis image of PCR product from S. Typhi hotspot parE with the expected fragments of 688 bp. Table S7. Electrophoresis of PCR product from S. Typhi hotspot parE with the expected fragments of 688 bp. Figure S6. Original electrophoresis image of PCR product from S. Typhi hotspot parE with the expected fragments of 688 bp. [file 12866_2022_2666_MOESM1_ESM.pdf]

Table S1. NCBI Genbank accession numbers and other information related to all hotspot sequences from *S.* Typhi isolates in Jakarta.

Isolate Number	Gene	Accession Number	Strain	Year	Location	Mutation	Susceptibility Profile			
							CIP	NAL	LVX	MXF
1	*gyr*A	ON220744	JKT-B2016-1	2016	Jakarta	No mutation	S	S	S	S
2	*gyr*A	ON220745	JKT-B2015-2	2015	Jakarta	No mutation	S	S	S	S
3	*gyr*A	ON220746	JKT-B2015-3	2015	Jakarta	No mutation	S	S	S	S
4	*gyr*A	ON220747	JKT-B2015-4	2015	Jakarta	No mutation	S	S	S	S
5	*gyr*A	ON220748	JKT-B2016-5	2016	Jakarta	No mutation	I	S	S	S
6	*gyr*A	ON220749	JKT-B2015-6	2015	Jakarta	No mutation	S	S	S	S
7	*gyr*A	ON220750	JKT-B2017-7	2017	Jakarta	No mutation	S	S	S	S
8	*gyr*A	ON220751	JKT-B2016-8	2016	Jakarta	No mutation	S	S	S	S
9	*gyr*A	ON220752	JKT-B2015-9	2015	Jakarta	No mutation	S	S	S	S
10	*gyr*A	ON220753	JKT-B2015-10	2015	Jakarta	No mutation	S	S	S	S
11	*gyr*A	ON220754	JKT-B2017-11	2017	Jakarta	No mutation	S	S	S	S
12	*gyr*A	ON220755	JKT-B2015-12	2015	Jakarta	No mutation	S	S	S	S
13	*gyr*A	ON220756	JKT-B2018-13	2018	Jakarta	No mutation	S	S	S	S
14	*gyr*A	ON220757	JKT-B2015-14	2015	Jakarta	No mutation	S	S	S	S
15	*gyr*A	ON220758	JKT-B2015-15	2015	Jakarta	No mutation	S	S	S	S

16	*gyr*A	ON220759	JKT-B2015-16	2015	Jakarta	No mutation	S	S	S	S
17	*gyr*A	ON220760	JKT-B2015-17	2015	Jakarta	No mutation	S	S	S	S
18	*gyr*A	ON220761	JKT-B2016-18	2016	Jakarta	No mutation	S	S	S	S
19	*gyr*A	ON220762	JKT-B2019-19	2019	Jakarta	No mutation	S	S	S	S
20	*gyr*A	ON220763	JKT-B2015-20	2015	Jakarta	No mutation	S	S	S	S
21	*gyr*A	ON220764	JKT-B2015-21	2015	Jakarta	No mutation	S	S	S	S
22	*gyr*A	ON220765	JKT-B2015-22	2015	Jakarta	No mutation	S	S	S	S
23	*gyr*A	ON220766	JKT-B2015-23	2015	Jakarta	No mutation	S	S	S	S
24	*gyr*A	ON220767	JKT-B2016-24	2016	Jakarta	No mutation	S	S	S	S
25	*gyr*A	ON220768	JKT-B2017-25	2017	Jakarta	No mutation	S	S	S	S
26	*gyr*A	ON220769	JKT-B2016-26	2016	Jakarta	No mutation	S	S	S	S
27	*gyr*A	ON220770	JKT-B2021-27	2021	Jakarta	No mutation	S	S	S	S
28	*gyr*A	ON220771	JKT-B2021-28	2021	Jakarta	No mutation	S	S	S	S
1	*gyr*B	ON220772	JKT-B2016-1	2016	Jakarta	No mutation	S	S	S	S
2	*gyr*B	ON220773	JKT-B2015-2	2015	Jakarta	No mutation	S	S	S	S
3	*gyr*B	ON220774	JKT-B2015-3	2015	Jakarta	No mutation	S	S	S	S
4	*gyr*B	ON220775	JKT-B2015-4	2015	Jakarta	No mutation	S	S	S	S
5	*gyr*B	ON220776	JKT-B2016-5	2016	Jakarta	No mutation	I	S	S	S
6	*gyr*B	ON220777	JKT-B2015-6	2015	Jakarta	No mutation	S	S	S	S

7	*gyr*B	ON220778	JKT-B2017-7	2017	Jakarta	No mutation	S	S	S	S
8	*gyr*B	ON220779	JKT-B2016-8	2016	Jakarta	No mutation	S	S	S	S
9	*gyr*B	ON220780	JKT-B2015-9	2015	Jakarta	No mutation	S	S	S	S
10	*gyr*B	ON220781	JKT-B2015-10	2015	Jakarta	No mutation	S	S	S	S
11	*gyr*B	ON220782	JKT-B2017-11	2017	Jakarta	No mutation	S	S	S	S
12	*gyr*B	ON220783	JKT-B2015-12	2015	Jakarta	No mutation	S	S	S	S
13	*gyr*B	ON220784	JKT-B2018-13	2018	Jakarta	No mutation	S	S	S	S
14	*gyr*B	ON220785	JKT-B2015-14	2015	Jakarta	No mutation	S	S	S	S
15	*gyr*B	ON220786	JKT-B2015-15	2015	Jakarta	No mutation	S	S	S	S
16	*gyr*B	ON220787	JKT-B2015-16	2015	Jakarta	No mutation	S	S	S	S
17	*gyr*B	ON220788	JKT-B2015-17	2015	Jakarta	No mutation	S	S	S	S
18	*gyr*B	ON220789	JKT-B2016-18	2016	Jakarta	No mutation	S	S	S	S
19	*gyr*B	ON220790	JKT-B2019-19	2019	Jakarta	No mutation	S	S	S	S
20	*gyr*B	ON220791	JKT-B2015-20	2015	Jakarta	No mutation	S	S	S	S
21	*gyr*B	ON220792	JKT-B2015-21	2015	Jakarta	No mutation	S	S	S	S
22	*gyr*B	ON220793	JKT-B2015-22	2015	Jakarta	No mutation	S	S	S	S
23	*gyr*B	ON220794	JKT-B2015-23	2015	Jakarta	No mutation	S	S	S	S
24	*gyr*B	ON220795	JKT-B2016-24	2016	Jakarta	No mutation	S	S	S	S
25	*gyr*B	ON220796	JKT-B2017-25	2017	Jakarta	No mutation	S	S	S	S

26	*gyr*B	ON220797	JKT-B2016-26	2016	Jakarta	No mutation	S	S	S	S
27	*gyr*B	ON220798	JKT-B2021-27	2021	Jakarta	No mutation	S	S	S	S
28	*gyr*B	ON220799	JKT-B2021-28	2021	Jakarta	No mutation	S	S	S	S
1	*par*C	ON220800	JKT-B2016-1	2016	Jakarta	No mutation	S	S	S	S
2	*par*C	ON220801	JKT-B2015-2	2015	Jakarta	No mutation	S	S	S	S
3	*par*C	ON220802	JKT-B2015-3	2015	Jakarta	No mutation	S	S	S	S
4	*par*C	ON220803	JKT-B2015-4	2015	Jakarta	No mutation	S	S	S	S
5	*par*C	ON220804	JKT-B2016-5	2016	Jakarta	No mutation	I	S	S	S
6	*par*C	ON220805	JKT-B2015-6	2015	Jakarta	No mutation	S	S	S	S
7	*par*C	ON220806	JKT-B2017-7	2017	Jakarta	No mutation	S	S	S	S
8	*par*C	ON220807	JKT-B2016-8	2016	Jakarta	No mutation	S	S	S	S
9	*par*C	ON220808	JKT-B2015-9	2015	Jakarta	No mutation	S	S	S	S
10	*par*C	ON220809	JKT-B2015-10	2015	Jakarta	No mutation	S	S	S	S
11	*par*C	ON220810	JKT-B2017-11	2017	Jakarta	No mutation	S	S	S	S
12	*par*C	ON220811	JKT-B2015-12	2015	Jakarta	No mutation	S	S	S	S
13	*par*C	ON220812	JKT-B2018-13	2018	Jakarta	No mutation	S	S	S	S
14	*par*C	ON220813	JKT-B2015-14	2015	Jakarta	No mutation	S	S	S	S
15	*par*C	ON220814	JKT-B2015-15	2015	Jakarta	No mutation	S	S	S	S
16	*par*C	ON220815	JKT-B2015-16	2015	Jakarta	No mutation	S	S	S	S

17	*par*C	ON220816	JKT-B2015-17	2015	Jakarta	No mutation	S	S	S	S
18	*par*C	ON220817	JKT-B2016-18	2016	Jakarta	No mutation	S	S	S	S
19	*par*C	ON220818	JKT-B2019-19	2019	Jakarta	No mutation	S	S	S	S
20	*par*C	ON220819	JKT-B2015-20	2015	Jakarta	No mutation	S	S	S	S
21	*par*C	ON220820	JKT-B2015-21	2015	Jakarta	No mutation	S	S	S	S
22	*par*C	ON220821	JKT-B2015-22	2015	Jakarta	No mutation	S	S	S	S
23	*par*C	ON220822	JKT-B2015-23	2015	Jakarta	No mutation	S	S	S	S
24	*par*C	ON220823	JKT-B2016-24	2016	Jakarta	No mutation	S	S	S	S
25	*par*C	ON220824	JKT-B2017-25	2017	Jakarta	No mutation	S	S	S	S
26	*par*C	ON220825	JKT-B2016-26	2016	Jakarta	No mutation	S	S	S	S
27	*par*C	ON220826	JKT-B2021-27	2021	Jakarta	No mutation	S	S	S	S
28	*par*C	ON220827	JKT-B2021-28	2021	Jakarta	No mutation	S	S	S	S
1	*par*E	ON220828	JKT-B2016-1	2016	Jakarta	No mutation	S	S	S	S
2	*par*E	ON220829	JKT-B2015-2	2015	Jakarta	No mutation	S	S	S	S
3	*par*E	ON220830	JKT-B2015-3	2015	Jakarta	No mutation	S	S	S	S
4	*par*E	ON220831	JKT-B2015-4	2015	Jakarta	No mutation	S	S	S	S
5	*par*E	ON220832	JKT-B2016-5	2016	Jakarta	No mutation	I	S	S	S
6	*par*E	ON220833	JKT-B2015-6	2015	Jakarta	No mutation	S	S	S	S
7	*par*E	ON220834	JKT-B2017-7	2017	Jakarta	No mutation	S	S	S	S

8	*par*E	ON220835	JKT-B2016-8	2016	Jakarta	No mutation	S	S	S	S
9	*par*E	ON220836	JKT-B2015-9	2015	Jakarta	No mutation	S	S	S	S
10	*par*E	ON220837	JKT-B2015-10	2015	Jakarta	No mutation	S	S	S	S
11	*par*E	ON220838	JKT-B2017-11	2017	Jakarta	No mutation	S	S	S	S
12	*par*E	ON220839	JKT-B2015-12	2015	Jakarta	No mutation	S	S	S	S
13	*par*E	ON220840	JKT-B2018-13	2018	Jakarta	No mutation	S	S	S	S
14	*par*E	ON220841	JKT-B2015-14	2015	Jakarta	No mutation	S	S	S	S
15	*par*E	ON220842	JKT-B2015-15	2015	Jakarta	No mutation	S	S	S	S
16	*par*E	ON220843	JKT-B2015-16	2015	Jakarta	No mutation	S	S	S	S
17	*par*E	ON220844	JKT-B2015-17	2015	Jakarta	No mutation	S	S	S	S
18	*par*E	ON220845	JKT-B2016-18	2016	Jakarta	No mutation	S	S	S	S
19	*par*E	ON220846	JKT-B2019-19	2019	Jakarta	No mutation	S	S	S	S
20	*par*E	ON220847	JKT-B2015-20	2015	Jakarta	No mutation	S	S	S	S
21	*par*E	ON220848	JKT-B2015-21	2015	Jakarta	No mutation	S	S	S	S
22	*par*E	ON220849	JKT-B2015-22	2015	Jakarta	No mutation	S	S	S	S
23	*par*E	ON220850	JKT-B2015-23	2015	Jakarta	No mutation	S	S	S	S
24	*par*E	ON220851	JKT-B2016-24	2016	Jakarta	No mutation	S	S	S	S
25	*par*E	ON220852	JKT-B2017-25	2017	Jakarta	No mutation	S	S	S	S
26	*par*E	ON220853	JKT-B2016-26	2016	Jakarta	No mutation	S	S	S	S

27	*par*E	ON220854	JKT-B2021-27	2021	Jakarta	No mutation	S	S	S	S
28	*par*E	ON220855	JKT-B2021-28	2021	Jakarta	No mutation	S	S	S	S

Table S2. Electrophoresis of PCR product from *S.* Typhi hotspot *gyr*A and *gyr*B with the expected fragments of 381 bp and 513 bp, respectively.

Lane #	SampleName	SamType	SamSize	Volume	Progress	Comments	Following Action
1	1A	Unpurified PCR Product	381	20	Proceed	PCR clean up.	
2	19A	Unpurified PCR Product	381	20	Proceed	PCR clean up.	
3	1B	Unpurified PCR Product	513	20	Proceed	PCR clean up.	
4	19B	Unpurified PCR Product	513	20	Proceed	PCR clean up.	

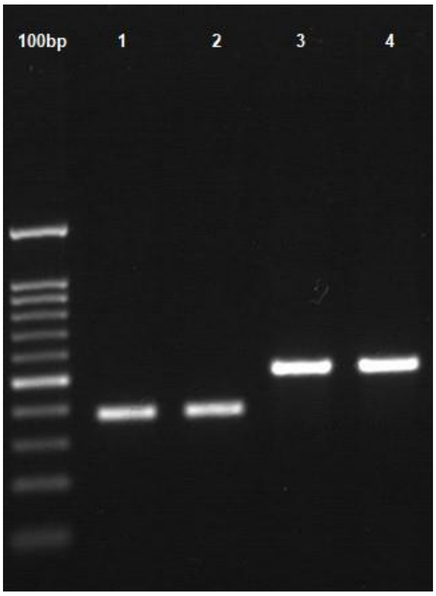

Condition:			0.8%	agarose gel							
Amount of DNA ladder loaded per lane:			0.1ug	each							
Volume of sample loaded per lane:			1uL	each							
**100bp DNA Ladder (bp):**	100	200	300	400	**500**	600	700	800	900	1,000	**1,500**
**100bp DNA Ladder (ng/0.1ug):**	8	8	7	8	**17**	6	5.5	8	8	10	**14.5**

Note: The DNA ladder is not applicable for sizing comparison of non-linear DNA samples (e.g. plasmid DNA)

Figure S1. Original electrophoresis image of PCR product from *S.* Typhi hotspot *gyr*A and *gyr*B with the expected fragments of 381 bp and 513 bp, respectively.

Table S3. Electrophoresis of PCR product from *S.* Typhi hotspot *gyr*A and *gyr*B with the expected fragments of 381 bp and 513 bp, respectively.

Rank	SampleID	OrderID	S.Name	S.Type	S.Size	CommentsAQ	AQ Status	SuggestionAQ
1	2701867	202051	3A	Unpurified PCR Product	381	PCR clean up.	PASS	
2	2701868	202051	10A	Unpurified PCR Product	381	PCR clean up.	PASS	
3	2701869	202051	16A	Unpurified PCR Product	381	PCR clean up.	PASS	
4	2701870	202051	22A	Unpurified PCR Product	381	PCR clean up.	PASS	
5	2701871	202051	25A	Unpurified PCR Product	381	PCR clean up.	PASS	
6	2701872	202051	26A	Unpurified PCR Product	381	PCR clean up.	PASS	
7	2701873	202051	28A	Unpurified PCR Product	381	PCR clean up.	PASS	
8	2701874	202051	32A	Unpurified PCR Product	381	PCR clean up.	PASS	
9	2701875	202051	34A	Unpurified PCR Product	381	PCR clean up.	PASS	
10	2701876	202051	36A	Unpurified PCR Product	381	PCR clean up.	PASS	
11	2701877	202051	38A	Unpurified PCR Product	381	PCR clean up.	PASS	
12	2701878	202051	39A	Unpurified PCR Product	381	PCR clean up.	PASS	
13	2701879	202051	40A	Unpurified PCR Product	381	PCR clean up.	PASS	
14	2701880	202051	44A	Unpurified PCR Product	381	PCR clean up.	PASS	
15	2701881	202051	48A	Unpurified PCR Product	381	PCR clean up.	PASS	
16	2701882	202051	52A	Unpurified PCR Product	381	PCR clean up.	PASS	
17	2701883	202051	55A	Unpurified PCR Product	381	PCR clean up.	PASS	
18	2701884	202051	57A	Unpurified PCR Product	381	PCR clean up.	PASS	
19	2701885	202051	59A	Unpurified PCR Product	381	PCR clean up.	PASS	
20	2701886	202051	60A	Unpurified PCR Product	381	PCR clean up.	PASS	
21	2701887	202051	61A	Unpurified PCR Product	381	PCR clean up.	PASS	
22	2701888	202051	62A	Unpurified PCR Product	381	PCR clean up.	PASS	
23	2701889	202051	96A	Unpurified PCR Product	381	PCR clean up.	PASS	
24	2701890	202051	30A	Unpurified PCR Product	381	PCR clean up.	PASS	
25	2701891	202051	AA	Unpurified PCR Product	381	PCR clean up.	PASS	
26	2701892	202051	A2A	Unpurified PCR Product	381	PCR clean up.	PASS	
27	2701893	202051	3B	Unpurified PCR Product	513	PCR clean up.	PASS	
28	2701894	202051	10B	Unpurified PCR Product	513	PCR clean up.	PASS	
29	2701895	202051	16B	Unpurified PCR Product	513	PCR clean up.	PASS	
30	2701896	202051	22B	Unpurified PCR Product	513	PCR clean up.	PASS	
31	2701897	202051	25B	Unpurified PCR Product	513	PCR clean up.	PASS	
32	2701898	202051	26B	Unpurified PCR Product	513	PCR clean up.	PASS	
33	2701899	202051	28B	Unpurified PCR Product	513	PCR clean up.	PASS	
34	2701900	202051	32B	Unpurified PCR Product	513	PCR clean up.	PASS	
35	2701901	202051	34B	Unpurified PCR Product	513	PCR clean up.	PASS	
36	2701902	202051	36B	Unpurified PCR Product	513	PCR clean up.	PASS	
37	2701903	202051	38B	Unpurified PCR Product	513	PCR clean up.	PASS	
38	2701904	202051	39B	Unpurified PCR Product	513	PCR clean up.	PASS	
39	2701905	202051	40B	Unpurified PCR Product	513	PCR clean up.	PASS	
40	2701906	202051	44B	Unpurified PCR Product	513	PCR clean up.	PASS	
41	2701907	202051	48B	Unpurified PCR Product	513	PCR clean up.	PASS	
42	2701908	202051	52B	Unpurified PCR Product	513	PCR clean up.	PASS	
43	2701909	202051	55B	Unpurified PCR Product	513	PCR clean up.	PASS	
44	2701910	202051	57B	Unpurified PCR Product	513	PCR clean up.	PASS	
45	2701911	202051	59B	Unpurified PCR Product	513	PCR clean up.	PASS	
46	2701912	202051	60B	Unpurified PCR Product	513	PCR clean up.	PASS	
47	2701913	202051	61B	Unpurified PCR Product	513	PCR clean up.	PASS	
48	2701914	202051	62B	Unpurified PCR Product	513	PCR clean up.	PASS	
49	2701915	202051	96B	Unpurified PCR Product	513	PCR clean up.	PASS	
50	2701916	202051	30B	Unpurified PCR Product	513	PCR clean up.	PASS	
51	2701917	202051	AB	Unpurified PCR Product	513	PCR clean up.	PASS	
52	2701918	202051	A2B	Unpurified PCR Product	513	PCR clean up.	PASS	

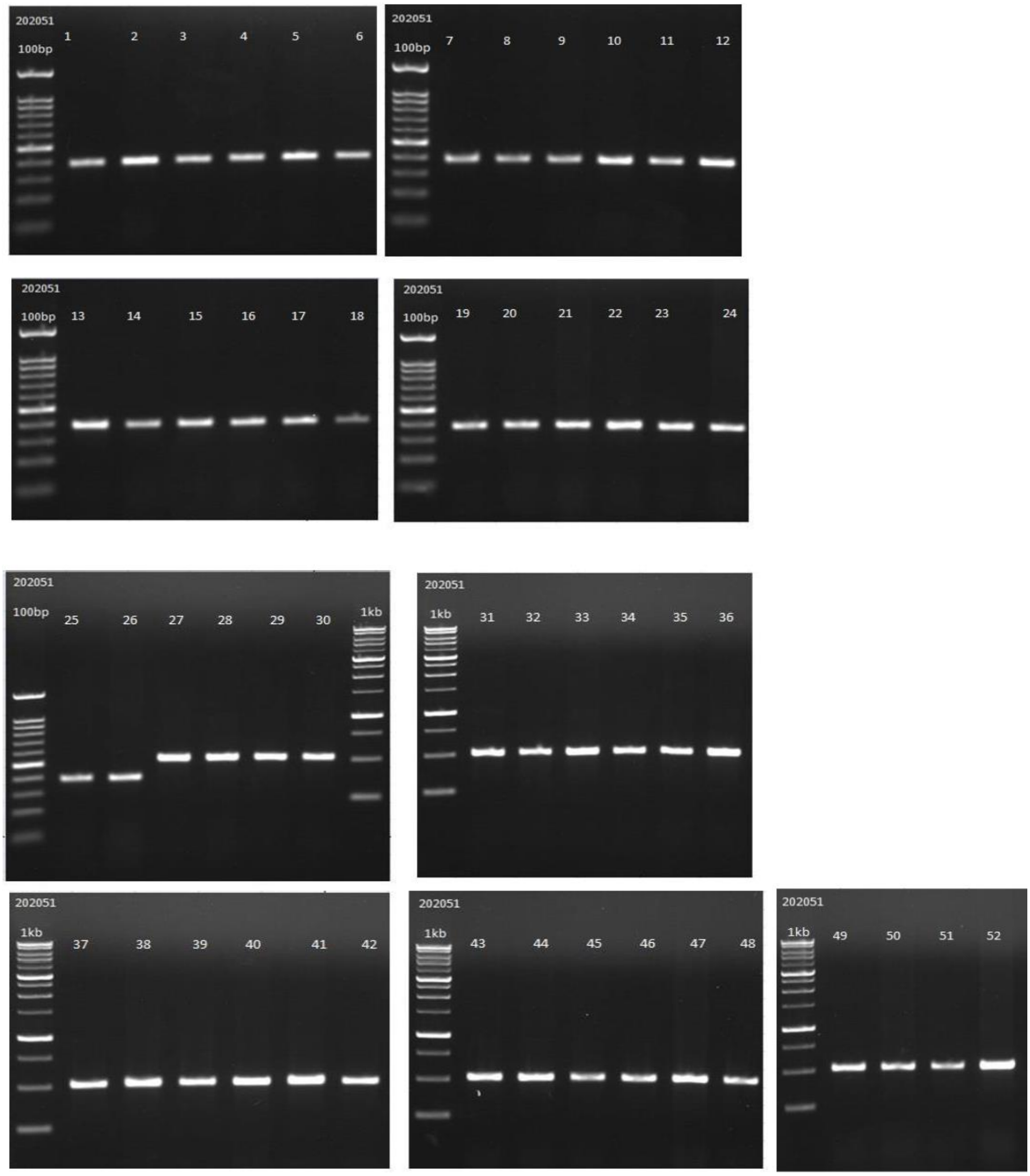

Condition:			0.8%	agarose gel									
Amount of DNA ladder loaded per lane:			0.1ug	each									
Volume of sample loaded per lane:			1uL	each									
**1kb DNA Ladder (bp):**	250	500	750	**1,000**	1,500	2,000	2,500	**3,000**	4,000	5,000	6,000	8,000	10,000
**1kb DNA Ladder (ng/0.1ug):**	9	6	4.6	18.4	4	6.8	6.8	**18.4**	3.6	5.6	5.6	5.6	5.6

Note: The DNA ladder is not applicable for sizing comparison of non-linear DNA samples (e.g. plasmid DNA)

Condition:			0.8%	agarose gel							
Amount of DNA ladder loaded per lane:			0.1ug	each							
Volume of sample loaded per lane:			1uL	each							
**100bp DNA Ladder (bp):**	100	200	300	400	**500**	600	700	800	900	1,000	**1,500**
**100bp DNA Ladder (ng/0.1ug):**	8	8	7	8	**17**	6	5.5	8	8	10	**14.5**

Note: The DNA ladder is not applicable for sizing comparison of non-linear DNA samples (e.g. plasmid DNA)

Figure S2. Original electrophoresis image of PCR product from *S.* Typhi hotspot *gyr*A and *gyr*B with the expected fragments of 381 bp and 513 bp, respectively.

Table S4. Electrophoresis of PCR product from *S.* Typhi hotspot *par*C with the expected fragments of 564 bp.

Rank	SampleID	OrderID	S.Name	S.Type	S.Size	CommentsAQ	AQ Status	SuggestionAQ
18	2752126	205506	3C	Unpurified PCR Product	564	PCR CleanUp.	PASS	
19	2752127	205506	10C	Unpurified PCR Product	564	PCR CleanUp.	PASS	
20	2752128	205506	16C	Unpurified PCR Product	564	PCR CleanUp.	PASS	
21	2752129	205506	22C	Unpurified PCR Product	564	PCR CleanUp.	PASS	
22	2752130	205506	25C	Unpurified PCR Product	564	PCR CleanUp.	PASS	
23	2752131	205506	26C	Unpurified PCR Product	564	PCR CleanUp.	PASS	
24	2752132	205506	28C	Unpurified PCR Product	564	PCR CleanUp.	PASS	
25	2752133	205506	32C	Unpurified PCR Product	564	PCR CleanUp.	PASS	
26	2752134	205506	34C	Unpurified PCR Product	564	PCR CleanUp.	PASS	
27	2752135	205506	36C	Unpurified PCR Product	564	PCR CleanUp.	PASS	
28	2752136	205506	38C	Unpurified PCR Product	564	PCR CleanUp.	PASS	
29	2752137	205506	39C	Unpurified PCR Product	564	PCR CleanUp.	PASS	
30	2752138	205506	40C	Unpurified PCR Product	564	PCR CleanUp.	PASS	
31	2752139	205506	44C	Unpurified PCR Product	564	PCR CleanUp.	PASS	
32	2752140	205506	48C	Unpurified PCR Product	564	PCR CleanUp.	PASS	
33	2752141	205506	52C	Unpurified PCR Product	564	PCR CleanUp.	PASS	
34	2752142	205506	55C	Unpurified PCR Product	564	PCR CleanUp.	PASS	
35	2752143	205506	57C	Unpurified PCR Product	564	PCR CleanUp.	PASS	
36	2752144	205506	59C	Unpurified PCR Product	564	PCR CleanUp.	PASS	
37	2752145	205506	60C	Unpurified PCR Product	564	PCR CleanUp.	PASS	
38	2752146	205506	61C	Unpurified PCR Product	564	PCR CleanUp.	PASS	
39	2752147	205506	62C	Unpurified PCR Product	564	PCR CleanUp.	PASS	
40	2752148	205506	96C	Unpurified PCR Product	564	PCR CleanUp.	PASS	
41	2752149	205506	30C	Unpurified PCR Product	564	PCR CleanUp.	PASS	
42	2752150	205506	AC	Unpurified PCR Product	564	PCR CleanUp.	PASS	
43	2752151	205506	A2C	Unpurified PCR Product	564	PCR CleanUp.	PASS	

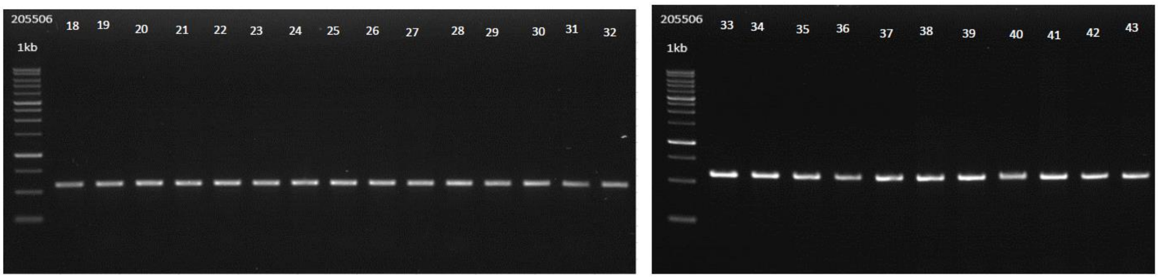

Condition:			0.8%	agarose gel									
Amount of DNA ladder loaded per lane:			0.1ug	each									
Volume of sample loaded per lane:			1uL	each									
**1kb DNA Ladder (bp):**	250	500	750	**1,000**	1,500	2,000	2,500	**3,000**	4,000	5,000	6,000	8,000	10,000
**1kb DNA Ladder (ng/0.1ug):**	9	6	4.6	18.4	4	6.8	6.8	**18.4**	3.6	5.6	5.6	5.6	5.6

Note: The DNA ladder is not applicable for sizing comparison of non-linear DNA samples (e.g. plasmid DNA)

Figure S3. Original electrophoresis image of PCR product from *S.* Typhi hotspot *par*C with the expected fragments of 564 bp.

Table S5. Electrophoresis of PCR product from *S.* Typhi hotspot *par*C with the expected fragments of 564 bp.

Rank	SampleID	OrderID	S.Name	S.Type	S.Size	CommentsAQ	AQ Status	SuggestionAQ
30	2739639	204744	1C	Unpurified PCR Product	564	PCR Cleanup.	PASS	
31	2739640	204744	19C	Unpurified PCR Product	564	PCR Cleanup.	PASS	

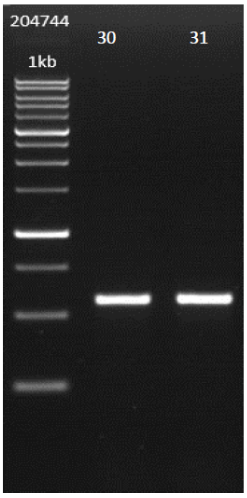

Condition: 0.8% agarose gel

Amount of DNA ladder loaded per lane: 0.1ug each

Volume of sample loaded per lane: 1uL each

**1kb DNA Ladder (bp):**	250	500	750	**1,000**	1,500	2,000	2,500	**3,000**	4,000	5,000	6,000	8,000	10,000
**1kb DNA Ladder (ng/0.1ug):**	9	6	4.6	18.4	4	6.8	6.8	**18.4**	3.6	5.6	5.6	5.6	5.6

Note: The DNA ladder is not applicable for sizing comparison of non-linear DNA samples (e.g. plasmid DNA)

Figure S4. Original electrophoresis image of PCR product from *S.* Typhi hotspot *par*C with the expected fragments of 564 bp.

Table S6. Electrophoresis of PCR product from *S.* Typhi hotspot *par*E with the expected fragments of 688 bp.

Rank	SampleID	OrderID	S.Name	S.Type	S.Size	CommentsAQ	AQ Status	SuggestionAQ
28	2739637	204743	1E	Unpurified PCR Product	688	No band.	FAIL	PCR clean up. Yield is not guaranteed. Result is not guaranteed.
29	2739638	204743	19E	Unpurified PCR Product	688	PCR Cleanup.	PASS	

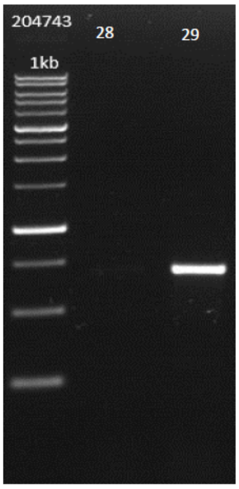

Condition:				0.8%	agarose gel									
Amount of DNA ladder loaded per lane:				0.1ug	each									
Volume of sample loaded per lane:				1uL	each									
**1kb DNA Ladder (bp):**	250	500	750	**1,000**	1,500	2,000	2,500	**3,000**	4,000	5,000	6,000	8,000	10,000	
**1kb DNA Ladder (ng/0.1ug):**	9	6	4.6	18.4	4	6.8	6.8	**18.4**	3.6	5.6	5.6	5.6	5.6	

Note: The DNA ladder is not applicable for sizing comparison of non-linear DNA samples (e.g. plasmid DNA)

Figure S5. Original electrophoresis image of PCR product from *S.* Typhi hotspot *par*E with the expected fragments of 688 bp.

Table S7. Electrophoresis of PCR product from *S.* Typhi hotspot *par*E with the expected fragments of 688 bp.

Rank	SampleID	OrderID	S.Name	S.Type	S.Size	CommentsAQ	AQ Status	SuggestionAQ
44	2752176	205508	1E	Unpurified PCR Product	688	PCR CleanUp.	PASS	
45	2752177	205508	3E	Unpurified PCR Product	688	PCR CleanUp.	PASS	
46	2752178	205508	10E	Unpurified PCR Product	688	PCR CleanUp.	PASS	
47	2752179	205508	16E	Unpurified PCR Product	688	PCR CleanUp.	PASS	
48	2752180	205508	22E	Unpurified PCR Product	688	PCR CleanUp.	PASS	
49	2752181	205508	25E	Unpurified PCR Product	688	PCR CleanUp.	PASS	
50	2752182	205508	26E	Unpurified PCR Product	688	PCR CleanUp.	PASS	
51	2752183	205508	28E	Unpurified PCR Product	688	PCR CleanUp.	PASS	
52	2752184	205508	32E	Unpurified PCR Product	688	PCR CleanUp.	PASS	
53	2752185	205508	34E	Unpurified PCR Product	688	PCR CleanUp.	PASS	
54	2752186	205508	36E	Unpurified PCR Product	688	PCR CleanUp.	PASS	
55	2752187	205508	38E	Unpurified PCR Product	688	PCR CleanUp.	PASS	
56	2752188	205508	39E	Unpurified PCR Product	688	PCR CleanUp.	PASS	
57	2752189	205508	40E	Unpurified PCR Product	688	PCR CleanUp.	PASS	
58	2752190	205508	44E	Unpurified PCR Product	688	PCR CleanUp.	PASS	
59	2752191	205508	48E	Unpurified PCR Product	688	PCR CleanUp.	PASS	
60	2752192	205508	52E	Unpurified PCR Product	688	PCR CleanUp.	PASS	
61	2752193	205508	55E	Unpurified PCR Product	688	PCR CleanUp.	PASS	
62	2752194	205508	57E	Unpurified PCR Product	688	PCR CleanUp.	PASS	
63	2752195	205508	59E	Unpurified PCR Product	688	PCR CleanUp.	PASS	
64	2752196	205508	60E	Unpurified PCR Product	688	PCR CleanUp.	PASS	
65	2752197	205508	61E	Unpurified PCR Product	688	PCR CleanUp.	PASS	
66	2752198	205508	62E	Unpurified PCR Product	688	PCR CleanUp.	PASS	
67	2752199	205508	96E	Unpurified PCR Product	688	PCR CleanUp.	PASS	
68	2752200	205508	30E	Unpurified PCR Product	688	PCR CleanUp.	PASS	
69	2752201	205508	AE	Unpurified PCR Product	688	PCR CleanUp.	PASS	
70	2752202	205508	A2E	Unpurified PCR Product	688	PCR CleanUp.	PASS	

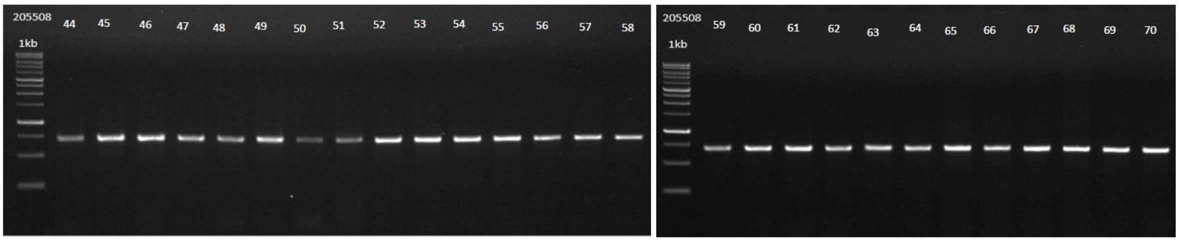

Condition:		0.8%	agarose gel										
Amount of DNA ladder loaded per lane:		0.1ug	each										
Volume of sample loaded per lane:		1uL	each										
**1kb DNA Ladder (bp):**	250	500	750	**1,000**	1,500	2,000	2,500	**3,000**	4,000	5,000	6,000	8,000	10,000
**1kb DNA Ladder (ng/0.1ug):**	9	6	4.6	18.4	4	6.8	6.8	**18.4**	3.6	5.6	5.6	5.6	5.6

Note: The DNA ladder is not applicable for sizing comparison of non-linear DNA samples (e.g. plasmid DNA)

Figure S6. Original electrophoresis image of PCR product from *S.* Typhi hotspot *par*E with the expected fragments of 688 bp.
